# Engineered nanomedicine triggering ROS storms for enhanced cancer therapeutics

**DOI:** 10.1093/rb/rbag029

**Published:** 2026-03-02

**Authors:** Yuebing Hou, Xiaoxuan Hou, Yiyao Wang, Chu Chu, Liya Tian, Nengyi Ni, Peiji Song, Yuchun Wei, Xiao Sun

**Affiliations:** Shandong Cancer Hospital and Institute, Shandong First Medical University and Shandong Academy of Medical Sciences, Jinan 250117, China; Shandong Cancer Hospital and Institute, Shandong First Medical University and Shandong Academy of Medical Sciences, Jinan 250117, China; Shandong Cancer Hospital and Institute, Shandong First Medical University and Shandong Academy of Medical Sciences, Jinan 250117, China; Department of Radiology, Central Hospital Affiliated to Shandong First Medical University, Jinan 250013, China; Shandong Cancer Hospital and Institute, Shandong First Medical University and Shandong Academy of Medical Sciences, Jinan 250117, China; Shandong Cancer Hospital and Institute, Shandong First Medical University and Shandong Academy of Medical Sciences, Jinan 250117, China; Department of Radiology, Central Hospital Affiliated to Shandong First Medical University, Jinan 250013, China; Shandong Cancer Hospital and Institute, Shandong First Medical University and Shandong Academy of Medical Sciences, Jinan 250117, China; Shandong Cancer Hospital and Institute, Shandong First Medical University and Shandong Academy of Medical Sciences, Jinan 250117, China

**Keywords:** ROS storm, oxidative stress, nanomedicine, cancer treatment

## Abstract

Reactive oxygen species (ROS) play a significant role in regulating various signaling pathways and biochemical reactions within cells during their proliferation and differentiation. Nonetheless, when excessive ROS are accumulated within cells, they will cause severe damage to cellular components like DNA, proteins and lipids. With advances in nanotechnology, ‘ROS storm’ has become a promising multifaceted therapeutic strategy for tumor treatment, whose mechanism mainly lies in inducing a massive ROS surge within tumors via synthesis optimization, the modulation of tumor adaptive responses and overcoming inherent constraints of individual ROS, so as to exceed the fatal threshold, thereby leading to severe oxidative damage. Therefore, in this review, we would like to focus on the specific strategies of engineered nanomaterials for triggering ROS storms in tumor tissues and the resultant effects, which have been primarily described in terms of augmenting substrate supply, inhibiting antioxidant systems, enhancing catalytic efficiency, enriching the diversity of reactive species and intensifying targeting accumulation, respectively. Moreover, the strong potential of combining this strategy with therapies such as immunotherapy has been demonstrated in various studies and constitutes a key focus of our discussion. At the end of the review, the future outlook and the remaining challenges when it comes to clinical application have also been discussed. This review is aimed at providing an overview of previous anti-cancer studies based on ROS storm, and shedding new light on this innovative strategy with fresh perspectives, thereby facilitating advances in cancer nanomedicine.

## Graphical abstract

**Graphical Abstract:**
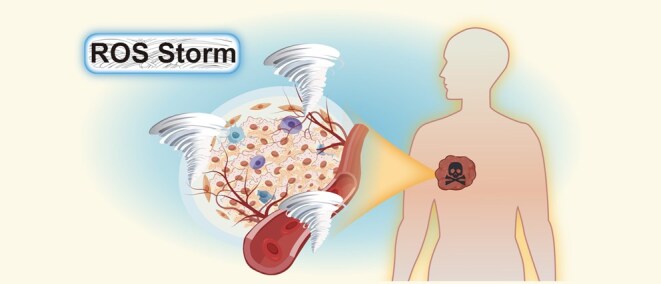

## Introduction

### Redox homeostasis

Reactive oxygen species (ROS) refer to oxygen-containing substances with active oxidizing characteristics in the body, including free radicals such as superoxide anions (•$O_{2}^{-}$), hydroxyl radicals (•OH) and nonradicals such as hydrogen peroxide (H_2_O_2_). Mostly, ROS at the physical level plays a significant role in regulating various signaling pathways, biochemical reactions within cells and cell proliferation and differentiation [1]. Nonetheless, excessive ROS will cause severe damage to cellular components such as DNA, proteins and lipids, causing even cell death under certain conditions [2]. There are ROS generation systems and antioxidant systems maintaining the balance of redox status, which is the critical foundation to ensure the functioning of cells. ROS generation systems mainly include the mitochondria, endoplasmic reticulum (ER) and peroxisome, where most ROS generation takes place during various redox reactions. On the other hand, the antioxidant system works by salvaging excess ROS in cells, mainly via enzymatic and non-enzymatic antioxidants such as superoxide dismutase (SOD1, SOD2 and SOD3), catalase (CAT), glutaredoxin, thioredoxin, reduced coenzyme Q (CoQH_2_) and bilirubin [3]. The disruption of redox state has significant relevance to many diseases [4]. For instance, the abnormal redox status can often be observed within tumor cells. Owing to a tremendous need for energy for proliferation, tumors are in an aberrantly hypermetabolic state, where ROS are overproduced and subsequently accumulated within cells. Accordingly, the level of ROS scavengers in tumor cells is relatively high in order to counteract oxidative stress. Therefore, an abnormal redox homeostasis is formed where the oxidizing and reducing substances are both at a high level, which greatly promotes the occurrence and progression of tumors and enhances their antioxidant defense [5]. This contributes to enormous obstacles in cancer therapy, which brings out the importance of breaking the redox homeostasis of tumor cells.

Substantial strategies have been dedicated to disrupting this abnormal redox homeostasis. Among them, increasing the accumulation of excessive ROS to kill cancer cells is a promising strategy that has been widely utilized in oncotherapy. However, if the ROS damage is just close to the lethal threshold but not beyond it, it is more likely to accelerate the tumor progression instead, suggesting that the key point of this strategy lies in achieving robust ROS destruction to actually exceed the threshold of tumor cells [6]. Unfortunately, most of the current ROS-based therapeutic strategies suffer from suboptimal performance. These strategies focus on simply increasing ROS quantity, which is restricted by shortcomings of ROS, such as the short half-life, limited diffusion distance and distribution within cells. Additionally, the complicated role of ROS in biological activity such as metabolism, signaling pathway and tumor microenvironment (TME) formation in tumors is another factor that affects tumor response to this ROS-mediated therapy. To comprehensively overcome these barriers, the concept of the ‘ROS storm’ has been proposed [7]. The ROS storm refers to a prospective strategy for oncotherapy that completely disrupts redox homeostasis to induce irreversible cell damage and even death by ROS synthesis optimization, the modulation of tumor adaptive responses and overcoming limitations in ROS intrinsic properties. With the rapid development of nanotechnology, various nanomaterials have promoted innovative advances in cancer treatment based on oxidative stress, making it feasible to achieve the induction of potent ROS storms [8, 9].

### The effects and mechanisms of oxidative damage the ROS storm causes to different positions in tumors

The ROS storm can cause oxidative harm to different locations in tumor cells by inducing the chemical modifications of DNA, proteins and lipids, mainly including the cell nucleus, mitochondria, ER, lysosomes, endosomes and cell membrane [10]. In terms of mitochondria, ROS can lead to protein-crosslinking and the formation of protein aggregates, thereby increasing the viscosity in the mitochondrial environment. This change of the microenvironment will limit the biomolecular interactions and metabolite diffusion, disrupting the respiration and metabolism of mitochondria [11, 12]. Besides, the lipids and proteins of the mitochondrial membrane can be oxidized by ROS, which elevates the mitochondrial permeability, triggering a series of reactions including the release of components such as cytochrome C and Ca^2+^ inside, mitochondrial depolarization, morphology change and its bioenergetic dysfunction [13].

In the ER, the main site for protein processing, significant enzymes involved in protein folding can be oxidized, leading to the accumulation of substantial misfolded proteins, which is highly related to the activation of PERK/eIF2α pathway and the subsequent overexpression of ER stress-related protein (CHOP) [14]. Consequently, ER stress is elicited, which not only hinders its function of processing proteins but also results in the leakiness of molecules such as Ca^2+^, the main factor of calcium overload [15–17].

On top of that, the cell membrane is another location likely to be damaged by ROS through the peroxidation of polyunsaturated phospholipids on it. Lethal lipid peroxides (LPOs) accumulate to destroy the function and structure of the cell membrane, resulting in its rupture eventually [18]. Similarly, ROS can induce lysosomal membrane permeabilization, resulting in the destruction of the membranes of lysosomes and endosomes. More importantly, it can then lead to the release of protease inside, thus triggering programmed cell death. Beyond the proteases, the leakage of numerous loosely bound iron ions (II) produced in the degradation of iron-containing proteins may elicit the Fenton reaction within the cytoplasm, generating more ROS eventually [19].

In regard to the cell nucleus, the stability of the chromatin within it is crucial for the fate of cells. ROS can target diverse areas of DNA, including adenine, guanine, the sugar moiety and methyl groups. These assaults result in a spectrum of oxidative lesions, such as the abnormal gene expression, DNA intrastrand crosslinks, DNA–protein crosslinks, mismatched pairs with damaged bases, stalled DNA replication forks, clustered lesions and single- and double-strand breaks (SSB-DSB) [20]. Furthermore, the guanine-rich telomeres are particularly susceptible to oxidation, leading to telomere shortening and destabilization of the protective chromosomal-end capping, which ultimately compromises genomic integrity and accelerates cellular senescence or death [10].

Since the homeostasis of subcellular organelles is the solid foundation of the survival and proliferation of cancer cells, the above-mentioned oxidative damage may initiate apoptosis in tumor cells [21]. Other than apoptosis, there are several other forms of cell death driven by severe injuries of critical subcellular organelles like mitochondria and the ER. These include immunogenic cell death (ICD) [22], pyroptosis [23, 24] and ferroptosis [25] with the hallmark of phospholipid peroxidation, which can be commonly seen during ROS storm-based anti-cancer therapy.

In addition to direct cytotoxicity, ROS storms also demonstrate potent indirect tumor-killing effects by interfering with subcellular organelle functions, channel proteins and metabolic pathways, thereby suppressing tumor growth, metastasis and recurrence. For instance, ROS can inhibit protein synthesis in tumor cells by damaging DNA, RNA, and degrading ribosomes, thus suppressing tumor growth, metastasis and recurrence [26, 27]. It is worth mentioning that these two killing modalities are closely linked to each other. For instance, the direct killing can potentiate indirect strategies through TME remodeling [28]. ROS storm-induced direct cell death, particularly ICD, ferroptosis and STING pathway activation [25, 29], can trigger the release of tumor-associated antigens (TAAs), damage-associated molecular patterns (DAMPs) and IFN-β. The released substances can stimulate dendritic cell (DC) maturation and subsequent T-cell activation, effectively transforming an immunosuppressive TME into an immunoresponsive one, thus enhancing the efficacy of immunotherapy [30].

Conversely, indirect tumor-killing effects such as tumor inhibition can increase tumor sensitivity to other therapies for stronger direct tumor killing, which demonstrates synergistic effects of ROS storms with other tumor treatments (Table 1). For example, mitochondrial dysfunction caused by oxidative damage will decrease the ATP generation efficiency. Thus, due to the lack of abundant energy supply, this will downregulate the expression of heat shock proteins (HSPs), which usually protect tumor cells from high temperature [31]. Eventually, the inhibition of cell heat resistance can be achieved, increasing their vulnerability to photothermal therapy (PTT) [32, 33]. Furthermore, mounting evidence indicates that the efflux pumps in the plasma membrane of tumor cells can induce the extracellular efflux of anti-cancer drugs, a process that highly relies on sufficient ATP supply [10]. The same ATP deficit can thereby reverse multidrug resistance by incapacitating drug efflux pumps, making previously resistant cells vulnerable again to chemotherapeutic agents [34]. Additionally, the ROS-mediated disruption of lysosomes and endosomes can prevent the degradation of internalized therapeutic agents, ensuring drugs retain their bioactivity and efficacy [8].

**Table 2:** Various nanomaterials trigger ROS storms for cancer therapy.

ROS storm mechanism	Material	Structure	ROS generation pathway	Enhancing strategy	Types of ROS	Effect	Cell type	Ref.
Augment substrate supply	FTP@RBCM	Nanoshuttle	CDT, PDT	H_2_O_2_↑, O_2_↑	•OH, ^1^O_2_	ICD, apoptosis	Hep3B	[44]
	Fe-MOF@Ir/GOx	Fusiform	CDT, PDT, nanoenzyme	H_2_O_2_↑, O_2_↑	•OH, ^1^O_2_	Apoptosis	MCF-7	[45]
	RuCN@PEG	Nanosheet	PDT	O_2_↑	•OH, ^1^O_2_, •$O_{2}^{-}$	Apoptosis, paraptosis, ferroptosis, ICD	A375	[46]
	mCGYL-LOx	Yolk-like nanoparticle	CDT	H_2_O_2_↑	•OH	ICD	Renca	[47]
	Co-PN_3_-SA/CHO	Dodecahedron	CDT, nanoenzyme	H_2_O_2_↑	•OH, •$O_{2}^{-}$	Apoptosis, mitochondria damage	4T1	[48]
	Cu-DBCO/CL	Rod-like	Nanoenzyme	H_2_O_2_↑	•OH, •$O_{2}^{-}$	ICD, ferroptosis, accelerate cuproptosis	4T1	[49]
	Cu-NS@UK@POx	Mesoporous	Nanoenzyme	H_2_O_2_↑	•OH	Pyroptosis, ICD	4T1	[50]
	ZnO_2_@Ce6/CaP@CPPO/BSA	Nanosphere	PDT	H_2_O_2_↑	^1^O_2_, •$O_{2}^{-}$	Apoptosis, accelerate calcium overload	4T1	[51]
Inhibit antioxidant systems	TAF-HMON-CuP@PPDG	HMON	CDT	GSH depletion	•OH	Ferroptosis	4T1	[36]
	MAP NPs	Nanosphere	CDT	GSH depletion	•OH	Ferroptosis	4T1	[52]
	Fe/Se-CaP	Nanosphere	CDT, GSH catabolization	GSH depletion	•OH, •$O_{2}^{-}$	Apoptosis, DNA damage, produces tumor-associated antigens	B16-F10	[53]
	NaCl@ssss-VHMS	Hollow mesoporous shell	Mitochondria synthesis	GSH depletion	ROS	Ferroptosis, apoptosis	HepG2	[54]
	MS-275, V-9302, PEG2k-DSPE self-assembly NPs	Nanosphere	Mitochondria synthesis	Inhibit mTOR pathway, inhibit glutamine transporter protein SLC1A5	ROS	Pyroptosis	OCM-1	[55]
	HA-SRF/Ce6@HANPs	Rod-like	PDT	Inhibit system Xc- transporter	ROS	Apoptosis	4T1	[56]
	mFe@Psi	Nanoshuttle	SDT, CDT	Silence GPX4 gene	•OH, ^1^O_2_	Lysosomal membrane destruction, ferroptosis, ICD	K7M2, 143B	[57]
	RI@Z-P	Nanosphere	PDT	Silence Nrf2 gene	ROS	Mitochondria damage, lipid peroxidation, ICD, DNA damage activated STING pathway	B16-F10	[58]
	Fe_3_O_4_/CaP	Nanocube	CDT, mitochondria synthesis	Downregulate CoQH_2_	•OH, ^1^O_2_, •$O_{2}^{-}$	Mitochondria damage, ferroptosis, apoptosis	CT26	[59]
Enhance catalytic efficiency	PtMn nanocubes	Nanocube	CDT	Elevate local temperature	•OH	Cell death	Huh7	[60]
	pDF NAs	Nanosphere	CDT	Elevate local temperature	•OH	HSP90 downregulation, ferroptosis	4T1	[61]
	MnSiO_3_@DHA@CaCO_3_	Nanoflower	CDT	Regulate pH	•OH	Pyroptosis, apoptosis	4T1	[62]
	Zn80Co20-1000@P	Nanoflower	Nanoenzyme	Optimize catalyst property	•OH, •$O_{2}^{-}$	Apoptosis, ferroptosis	MCF-7/MDR	[6]
	O-Fe-N_4_	Nanosphere	Nanoenzyme	Optimize catalyst property	•OH, ^1^O_2_	Apoptosis, ferroptosis	4T1	[63]
	Fe/NV-CN	Flake	PDT	Optimize catalyst property	•OH	Apoptosis	Cal27	[64]
	UCNP/CeO_2_/CuO	Nanosphere	PDT, CDT	Optimize catalyst property	•OH, ^1^O_2_, •$O_{2}^{-}$	Apoptosis, ferroptosis	CT26	[65]
	MoS_2_-Cu_2_O-PEG@QE/Znpp IX	Nanoflower	CDT	Optimize catalyst property	•OH	Lysosomal membrane destruction, lysosomal cell death (LCD)	4T1	[66]
	Carrier-platin	Nanoparticle	CDT	Optimize catalyst property	•OH	DNA damage, lysosomal membrane destruction, ER damage, DNA induced cell death	CT26	[67]
Enrich the diversity of reactive species	FeCo/Fe-Co DAzyme/PL	Core-shell	Nanoenzyme	Multiple ROS	AA-OOH, •OH, ^1^O_2_, •$O_{2}^{-}$	Ferroptosis, ICD	4T1	[68]
	AsHMS-TA/FeIII@NK	Virus-like nanoparticle	CDT, radical generator	ROS, •C	•OH, •C	DNA double-strand break, apoptosis	HepG2	[69]
	AFeI FAND	Nanosphere	CDT, PDT, radical generator	ROS, •C	•C, •OH, ^1^O_2_, •$O_{2}^{-}$	DNA double-strand break, mitochondria damage, ferroptosis	4T1, Hela	[70]
	PNSO NPs	Nanoflower	Radical generator	ROS, •${\text{SO}}_{4}^{-}$	•OH, •${\text{SO}}_{4}^{-}$	Pyroptosis-ICD	4T1	[7]
	OH7	Nanogel	SDT, radical generator	ROS, RNS	ROO•, ONOO^−^, •OH, ^1^O_2_, •$O_{2}^{-}$	Apoptosis, ECM destruction	4T1	[71]
	Arg/Fc@GOx/HA	Nanosphere	CDT, radical generator	ROS, RNS	•OH, •$O_{2}^{-}$, NO, ONOO^−^	Cell death	A549	[72]
Intensify targeting accumulation	^CD44^FMNA	Core-shell	CDT, nanoenzyme	Biomarker targeting	•OH	Apoptosis	MDA-MB-231	[73]
	PDA/Cu/ICG/R	Nanosphere	CDT, PDT	Biomarker targeting	•OH, ^1^O_2_	HSP90 downregulation, apoptosis	4T1	[32]
	MFePCN@1-MT	Core-shell	SDT, CDT	Homologous targeting	•OH, ^1^O_2_	ICD, ferroptosis, DNA damage	K7M2	[74]
	TPP-HA-TDV NPs	Nanosphere	Radical generator	Organelle targeting	•C	ICD	H22, Hepa1-6	[75]

### Nanocatalytic therapy to effectively trigger strong ROS storms

In comparison with conventional ROS-based strategies, there exist specific conditions that are indispensable for triggering a dramatic ROS storm. A lethal level of generated ROS is undoubtedly pivotal, which can be achieved by the synergy of multiple ROS-yielding approaches [35] and the enhancement of each approach [36]. In terms of ROS generation improvement, it is imperative to implement comprehensive optimizations of the microenvironment where catalytic reactions take place, from the substrate magnification, suitable pH, to lowering intracellular antioxidant levels [36]. Meanwhile, highly efficient ROS generators are essential as well to induce the ROS storm. In addition to achieving adequate ROS synthesis, the long retention of substantial ROS stands as another significant factor, considering the short half-life of ROS [37].

The majority of strategies for ROS-based oncotherapy rely on catalytic reactions to yield ROS within cells, with or without physical stimuli such as light, ultrasound (US) and electricity. Most recent researches have centered around the methods to enhance the catalytic ROS generation reaction [38]. The progress made in nanotechnology has shed new light on this issue, thus inducing robust ROS storms. Nanomaterials have exhibited great potential owing to their unique intrinsic biophysical characteristics. On the one hand, with regard to ROS production, nanoscale drugs demonstrate distinctive catalytic activity and the ability to modulate the TME, which are attributed to larger surface area/volume and the controllability and adjustability of their structures and functions [39]. On the other hand, regarding the drug delivery platform, nanomaterials can passively target tumor tissues based on the enhanced permeability and retention (EPR) effect [40]. They have also been proven to improve the attributes of cancer drugs including the bioavailability, solubility and their behavior, such as pharmacokinetics [41]. Thereby, the therapeutic effects of drugs can be enhanced while minimizing side effects. Notably, the rational design of nanoplatforms is able to achieve co-loading of multiple drugs, effectively achieving synergistic strategies [42] and eliciting cascade reactions [43], thus augmenting the duration and intensity of ROS storms for cancer treatment.

**Figure 1:**
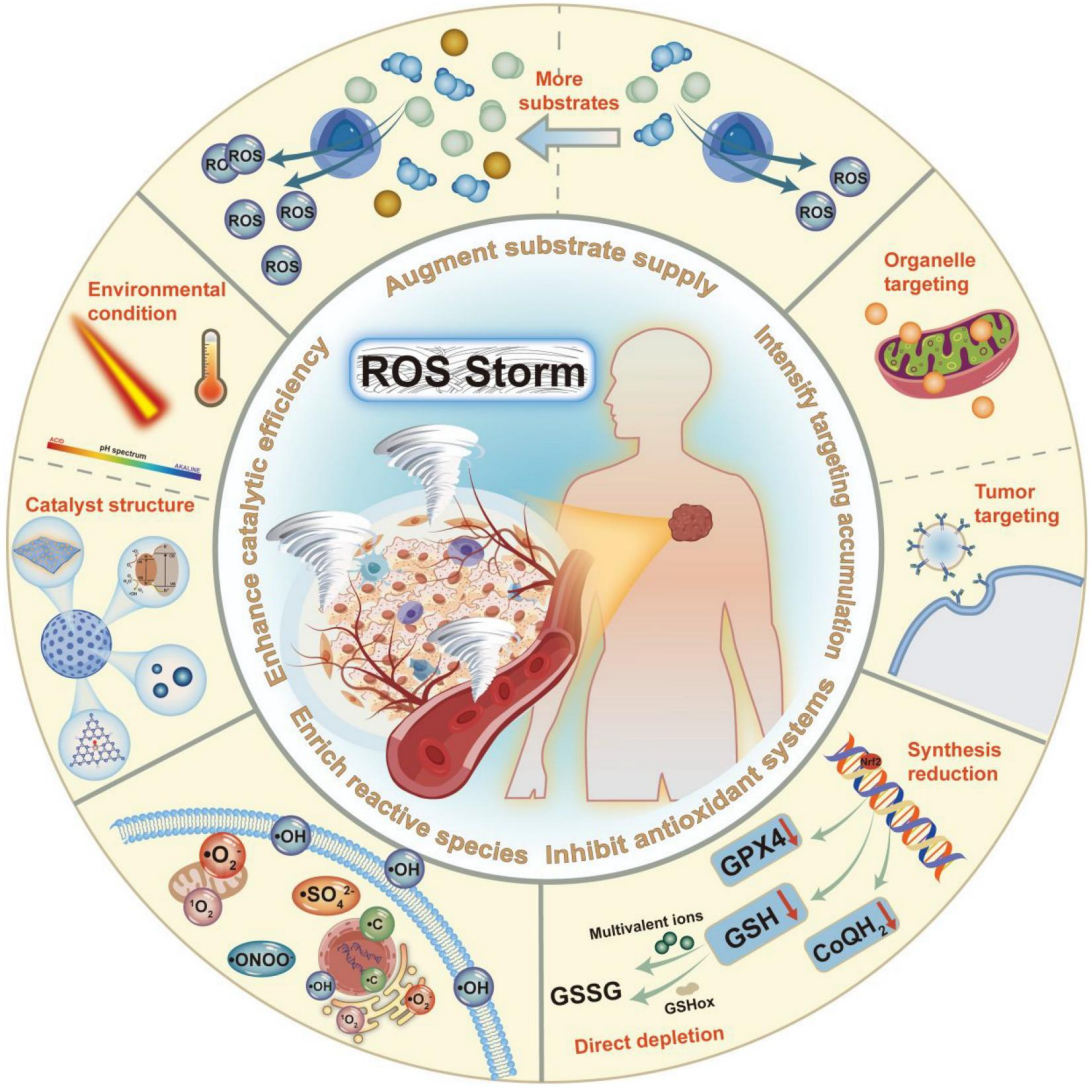
Schematic illustration of specific approaches to evoke ROS storms.

This review mainly introduces the latest advances in different nanotechnology-based strategies triggering ROS storms to achieve enhanced cancer treatment. The subsequent effects in tumor cells caused by ROS storms, and the mechanisms, specific ROS types have been summarized (Table 2). Specific approaches ranging from augmenting substrate supply, enhancing catalytic efficiency, enriching the diversity of ROS, to intensifying targeting accumulation of nanodrugs will be illustrated respectively (Figure 1).

**Table 1:** Representative nanomaterials for the synergistic therapy of ROS storms and other strategies.

Material	Structure	Synergistic therapy	Mechanism	Disease	Ref.
FTP@RBCM	MOFs	CDT, PDT and immunotherapy	ROS trigger ICD, ICB	Hepatic cancer	[44]
RuCN@PEG	Nanosheet	PDT and immunotherapy	ROS trigger ICD	Malignant melanoma	[46]
mCGYL-LOx	Yolk-like nanoparticle	CDT and immunotherapy, cuproptosis	PD-L1↓ (lactic acid depletion)	Clear cell renal cell carcinoma	[47]
Cu-DBCO/CL	Rod-like	Nanoenzyme and immunotherapy, cuproptosis	PD-L1, TIM-3↓ (cholesterol depletion) copper-transporting ATPase 1↓	Breast cancer	[49]
Cu-NS@UK@POx	Mesoporous	Nanoenzyme and immunotherapy	ROS trigger ICD, PD-L1↓ (lactic acid reduction)	Breast cancer	[50]
ZnO_2_@Ce6/CaP@CPPO/BSA	Nanosphere	PDT and immunotherapy, calcium overload	CTLA-4 blocking	Breast cancer	[51]
Fe/Se-CaP	Nanosphere	CDT, GSH catabolization and immunotherapy	Apoptosis produces tumor-associated antigens, PD-1 blocking	Melanoma	[53]
NaCl@ssss-VHMS	Hollow mesoporous shell	Ion-interference therapy	Osmolarity surge NaCl↑	Hepatic cancer	[54]
MS-275, V-9302, PEG2k-DSPE self-assembly NPs	Nanosphere	Chemotherapy and immunotherapy	ROS triggered pyroptosis releases IL-1β, IL-18	Uveal melanoma	[55]
mFe@Psi	Nanoshuttle	SDT, CDT and immunotherapy	ROS trigger ICD, DAMPs release	Breast cancer bone metastasis	[57]
RI@Z-P	Nanosphere	PDT and immunotherapy	cGAS/STING, IFN-β↑ mature DCs↑	Melanoma	[58]
MoS_2_-Cu_2_O-PEG@QE/Znpp IX	Nanoflower	CDT and PTT	HSP inhibitor	Breast cancer	[66]
MnSiO_3_@DHA@CaCO_3_	Core-shell	CDT and calcium overload	Calcium delivery	Breast cancer	[62]
FeCo/Fe-Co DAzyme/PL	Nanosphere	Nanoenzyme immunotherapy	ROS trigger ICD	Breast cancer	[68]
AsHMS-TA/FeIII@NK	Nanoflower	CDT, radical generator and PTT	PTT triggers •C release	Hepatic cancer	[69]
PNSO NPs	Virus-like nanoparticle	Radical generator, immunotherapy, calcium overload	ROS triggers pyroptosis, DAMPs release, CTLA-4 blocking	Breast cancer	[7]
PDA/Cu/ICG/R	Nanosphere	CDT, PDT and PTT	Oxidative stress downregulates HSP expression	Breast cancer	[32]
TPP-HA-TDV NPs	Core-shell	Radical generator and immunotherapy	ROS trigger ICD, mature DCs↑	Hepatic cancer	[75]
MFePCN@1-MT	Nanosphere	SDT, CDT and immunotherapy	ROS trigger ICD	Osteosarcoma	[74]

## Different nanomedicine-based strategies triggering ROS storms for enhanced cancer theranostics

### Augment substrate supply

Photodynamic therapy (PDT) is one of the ROS generation strategies that are commonly used in cancer treatment. It has been approved for several clinical treatments for lung cancer, skin cancer, esophageal cancer and others, with advantages of non-invasiveness and spatiotemporal controllability. In general, the nontoxic photosensitizers (PSs) activated by specific light will react with surrounding substrates to generate ROS such as ^1^O_2_ and •OH [76]. Similar to PDT, sonodynamic therapy (SDT) exploits US as the external stimulation to activate sonosensitizers. The interaction between the US and tissues induces the cavitation effect, which subsequently triggers chemical cascades for ROS generation [77].

Beyond PDT and SDT, chemodynamic therapy (CDT), a novel therapeutic strategy, has attracted much attention in recent years. Independent of external energy initiation, it can achieve ROS generation directly through endogenous Fenton or Fenton-like reactions, where the specific catalyzers convert H_2_O_2_ into •OH [78].

However, the above-mentioned modalities suffer from a common limitation, which is the reliability of the substrate concentration within cells. Unfortunately, a hypoxic TME is commonly seen in most solid cancers. Such a hypoxic environment impedes the behavior of oxygen-dependent therapies such as PDT by creating an acute substrate deficiency. Factors contributing to this include carcinogenic factors, high metabolism, nonfunctional angiogenesis, vascular pressure deformation and distant effect [41, 79]. Besides, the concentration of H_2_O_2_ in tumor cells is often insufficient to meet the need for the tremendous •OH generation in CDT [80]. Therefore, augmenting substrate supply would be a potential strategy to promote ROS storms by overcoming those limitations.

In recent developments, various nanomaterial-based modalities have been utilized to tackle the issues above, which can be categorized into two approaches: (i) achieving endogenous synthesis of substrates using components in tumor tissue [81]; (ii) delivering exogenous substrates to cancer cells with nanodrugs possessing substrate generating property [82]. Based on the former strategy, multiple studies have succeeded in improving O_2_ supply mainly through catalytic chemical reactions. Various nanoplatforms loaded with natural enzymes and emerging nanoenzymes that can mimic the properties of natural enzymes have made the modalities mentioned above accessible and effective [83].

**Figure 2:**
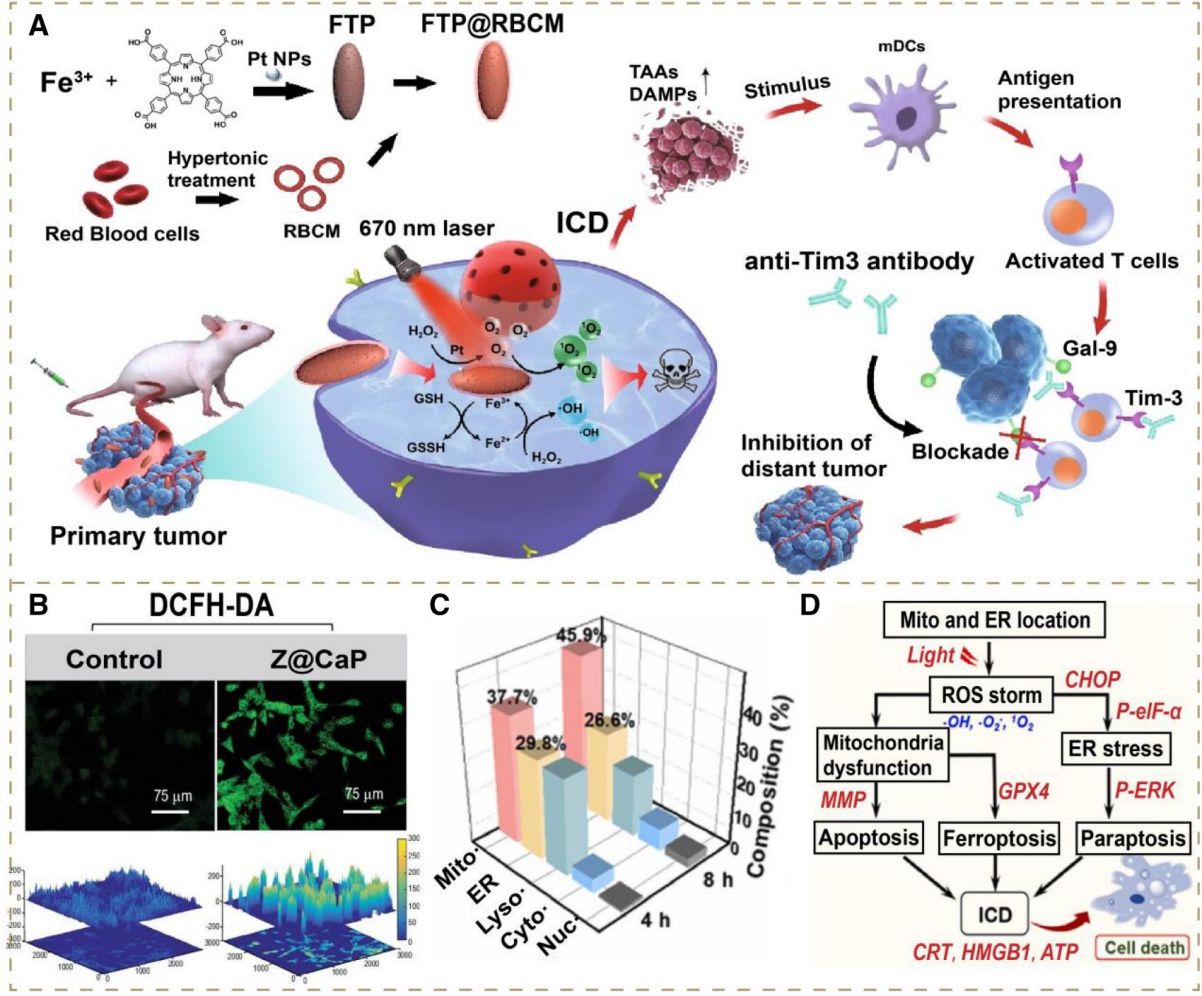
(**A**) Schematic illustrations of the preparation of artificial RBCs (FTP@RBCM) and the immunotherapeutic mechanisms of the combination of ROS storm-based therapy and Tim-3 checkpoint blockade. Reproduced with permission from Springer Nature Link, Copyright 2022 [44]. (**B**) Intracellular H_2_O_2_ generation measured by DCFH-DA. Reproduced with permission from Oxford University Press, Copyright 2021 [51]. (**C**) Time-dependent subcellular distribution of RuCN@PEG in A375 cells in the mitochondria, ER, lysosomes, cytoplasm and nucleus determined by ICP-MS. (**D**) Schematic illustration of the multimodal cell death mechanism of RuCN@PEG. Reproduced with permission from Elsevier Ltd, Copyright 2023 [46].

Among them, the conversion of H_2_O_2_ to O_2_ with CAT is widely used, which can produce considerable O_2_ to promote ^1^O_2_ synthesis. For example, Li *et al.* developed FTP@RBCM with the core of Fe-protoporphyrin-based hybrid metal-organic frameworks (FTP) doped with Pt nanoparticles (NPs) and an outer layer of RBCMs (Figure 2A). Pt NP doping endowed FTP with the activity of CAT, which could significantly alleviate local hypoxia. Subsequently, the photosensitizer porphyrin could generate a high level of ^1^O_2_ under 679 nm visible light with abundant O_2_, thus successfully triggering the ROS storm, leading to severe ICD of primary tumors under normoxic TME. The tumor-eliminating efficacy was evaluated via the CCK-8 assay, with cell viability declining below 80% at a TCPP concentration of 80 *μ*g mL^−1^. Remarkably, it still presented a superior PDT effect under a hypoxic environment with O_2_ self-supply capacity. Meanwhile, they blocked the T-cell immunoglobulin and mucin-containing molecule 3 (Tim-3) checkpoint, preventing its interaction with galectin-9 from mediating T-cell suppression. Eventually, this strategy could eliminate primary tumors while achieving distant tumor inhibition by stimulating immune responses through the synergistic treatment of ROS storm and immune checkpoint inhibitors (ICs) [44].

**Figure 3:**
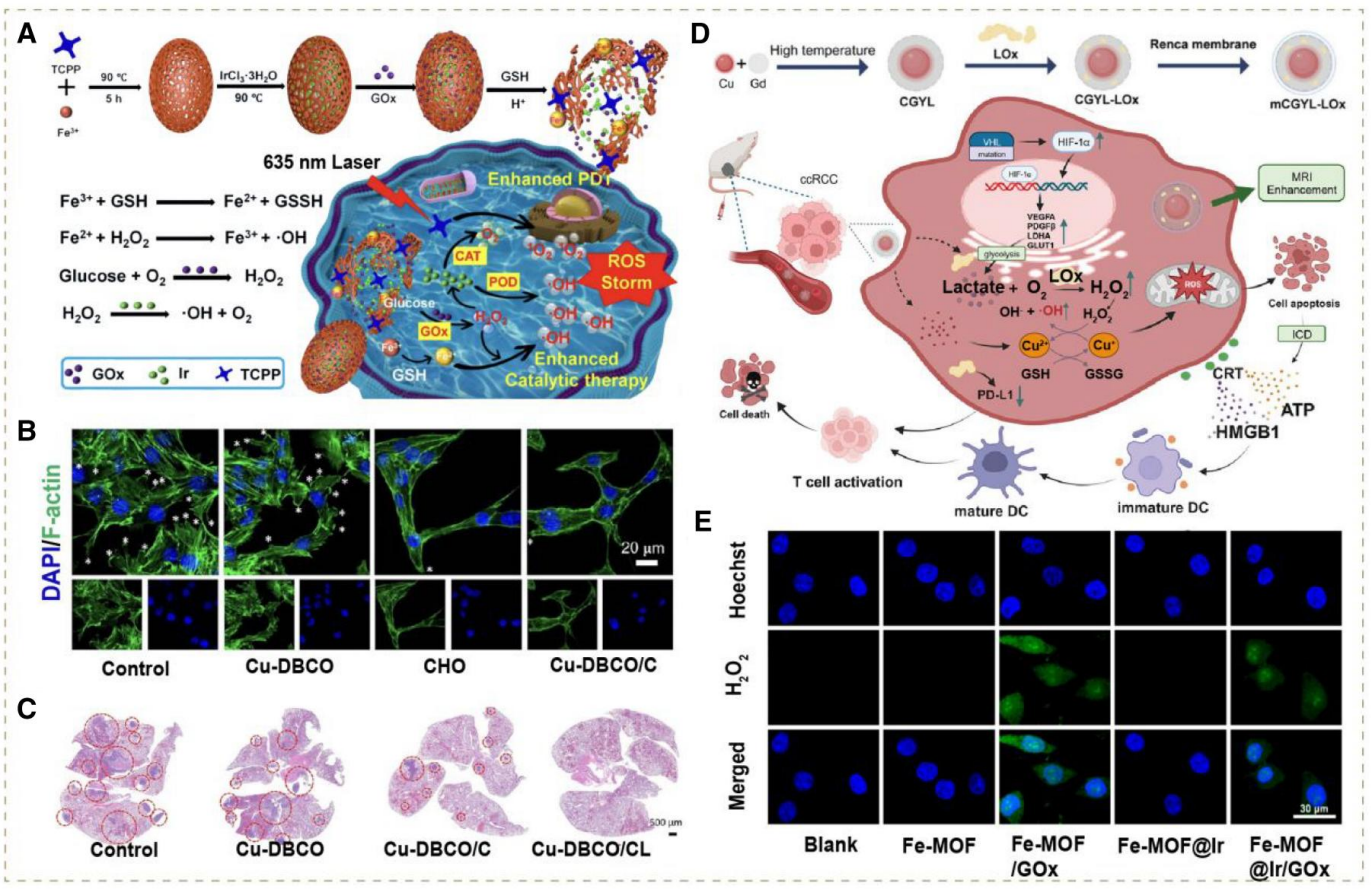
(**A**) Schematic illustrations of Fe-MOF@Ir/GOx nanoplatform with GOx/CAT/POD-like activities to strengthen PDT/catalytic therapy by producing ROS storms. Reproduced with permission from Elsevier, Copyright 2024 [45]. (**B**) Changes in lamellipodia and immunofluorescence of F-actin from the indicated treatment groups. Asterisk indicates lamellipodia. (**C**) H&E-stained images of lung tissues in different treatment groups. Reproduced with permission from American Chemical Society, Copyright 2024 [49]. (**D**) Schematic representation of the synthesis process of mCGYL-LOx and the anti-cancer mechanism of mCGYL-LOx induced cuproptosis, CDT and immunotherapy of tumor. Reproduced with permission from Wiley-VCH GmbH, Copyright 2024 [47]. (**E**) CLSM images of MCF-7 cells in different samples by detecting intracellular H_2_O_2_ generation. Reproduced with permission from Elsevier, Copyright 2024 [45].

Notably, the strategy above demonstrates the significance of sufficient H_2_O_2_ supply in augmenting O_2_ generation, let alone its huge demand as a substrate in •OH synthesis as well [41]. On these grounds, there is an urgent need to explore effective modalities to achieve high levels of H_2_O_2_ in tumor cells. One of the most extensively applied methodologies is to load glucose oxidase (GOx), which can reduce glucose into H_2_O_2_. In one such study, Zhang *et al.* designed an iron-coordinated porphyrin MOF (Fe-MOF) encapsulating ultrasmall iridium (Ir) and modifying GOx (Fe-MOF@Ir/GOx). The level of H_2_O_2_ within cells had been elevated by GOx. On the one hand, in synergy with the CAT-like Ir, massive O_2_ could be produced to amplify ^1^O_2_ synthesis in PDT; on the other hand, Ir possessed the activity of peroxidase (POD) as well, which was able to directly catalyze the abundant H_2_O_2_ into •OH together with Fenton catalyzer Fe^2+^. It was observed that by laser irradiation, the survival rate of cells incubated with Fe-MOF@Ir/GOx decreased to 5.59%, while without sufficient substrates, the survival rate of cells incubated with Fe-MOF was still 22.16% [45]. In summary, this strategy had effectively enhanced PDT/catalytic therapy (Figure 3A and E).

H_2_O is known as the most substantial compound in the organism, which has been utilized to generate oxygen for ROS-generating reactions via some nanomaterial-mediated catalysis. In comparison with H_2_O_2_, ample H_2_O can overcome the drawback of substrate deficiency in oxygen production. Moreover, the catalytic decomposition of H_2_O is activated by light stimuli, which means the process can be controlled spatiotemporally to elevate the biosafety and tumor-killing efficiency of drugs. Nevertheless, the efficacy relies on strong irradiation, which is often limited in deep-seated or large tumors. To tackle this issue, Wei’s group developed an oxygen-self-sufficient two-photon photosensitizer based on the coordination of Ru (II) polypyridine complexes to graphitic carbon nitride g-C_3_N_4_ nanosheets. The attachment of the metal complexes to the nanosheets remarkably reinforces two-photon absorption, surpassing two orders of magnitude observed in molecular Ru (II) polypyridine complexes. With potentiated stimulation, the photocatalysis mediated by g-C_3_N_4_ for higher O_2_ levels and the conversion of O_2_ into ROS can be strengthened. Eventually, the abundant ROS trigger powerful ROS storms in various subcellular locations (Figure 2C), leading to multiple paths of cell death (Figure 2D) [46].

Due to the aberrant metabolism, other components such as lactate are at high levels within tumor cells, which are related to progression and metastasis [84]. For these reasons, there are emerging strategies targeting these components. For instance, it has been shown by researchers that lactate is overproduced in clear cell renal cell carcinoma (ccRCC), which can improve proliferation and the formation of an immunosuppressive microenvironment. According to this, Xu *et al.* fabricated a novel Cu-based nanodrug (mCGYL-LOx), which loaded lactate oxidase (LOx) with ccRCC cell membrane camouflaging CuO@Gd_2_O_3_ yolk-like particles. The excessive amount of lactate could be converted into sufficient H_2_O_2_ through the oxidation of LOx, boosting more •OH production mediated by Fenton-like catalyst Cu^+^. An increase in the ROS level compared with drugs without LOx was measured by the DCFH-DA probe. Additionally, abundant Cu^+^ delivered by the nanodrug led to Cu^+^ overload in tumor cells, inducing cuproptosis, which, with the combination of ROS storm, remarkably activated severe ICD and the subsequent release of DAMPs. Notably, other than simply elevating substrate contents like aforementioned CAT, the lactate depletion via LOx could downregulate the expression of PD-L1 in tumors, which was validated through Western blot and quantitative polymerase chain reaction. This metabolism modulation reshaped the immunosuppressive TME significantly, further enhancing the immune response on the basis of ICD (Figure 3D) [47].

Additionally, cholesterol has also been reported to be retained at a high level in tumor tissue. Compared with lactate, cholesterol can not only support tumor proliferation as a resource of membrane biogenesis but also endow tumors with strong invasion and metastasis ability by inducing epithelial–mesenchymal transition. Liu and his co-workers constructed a phosphorus (P)-doped cobalt single-atom nanozyme loaded with cholesterol oxidase (Co-PN_3_ SA/CHO). In this process, the nanoenzyme, with its oxidase (OXD)-like and Fenton-like activities, could produce •$O_{2}^{-}$ and •OH by catalyzing O_2_ and H_2_O_2_, respectively. On this basis, the loaded cholesterol oxidase (CHO) possessed the ability to generate massive H_2_O_2_ by oxidizing excessive cholesterol accumulated in the TME, which synergized with CAT-like Co-PN_3_ SA to enhance substrate supply of H_2_O_2_ and O_2_ for the ROS-generating catalytic reactions mentioned above. Remarkably, the study also revealed that the depletion of cholesterol can significantly destroy the integrity of lipid rafts and suppress lamellipodia formation, thus inhibiting tumor proliferation and migration [48]. Another study conducted by Liu’s group confirmed that this form of cholesterol consumption modality can inhibit the formation of lamellipodia (Figure 3B) and downregulate the expression of ICs of T cells around tumors, such as PD-1 and TIM-3, thereby strengthening their infiltration. Consequently, a significant improvement in tumor eradication can be observed (Figure 3C) [49].

Pyruvate is known to participate in the metabolism of both anaerobic glycolysis and aerobic respiration as a critical substrate. It plays a central part in regulating the consequent lactate biosynthesis and energy supply, thus standing as a prospective target for tumor eradication as well. Inspired by this, Niu *et al.* prepared single-atom nanozymes loaded with pyruvate oxidase (POx) and mitochondrial pyruvate carrier (MPC) inhibitor UK5099 (Cu-NS@UK@POx). POx could oxidize excess intracellular pyruvate to abundant H_2_O_2_, thus facilitating Cu-NS with POD-like activity to trigger strong •OH storms by catalyzing H_2_O_2_. Aerobic respiration could be restricted by UK5099, which was able to prevent pyruvate from entering mitochondria, thus providing more oxygen for the POx-mediated redox reaction. In addition, metabolism disruption via the synergy of POx and MPC inhibitor could lead to tumor starvation and lactate downregulation. Collectively, the ROS storm in combination with pyruvate targeting could induce pyroptosis and reprogram the immunosuppressive TME [50]. Overall, the above strategies targeting endogenous substances of tumors could not only facilitate ROS storm induction but also interfere with cellular metabolism to achieve potent tumor killing and suppression of tumor progression simultaneously.

In the second subtype, one of the most typical strategies is applying nanomedicines carrying peroxide such as CaO_2_ and ZnO_2_, which further undergo decomposition in the TME to release large amounts of exogenous H_2_O_2_ [85]. For instance, Huang and co-workers designed a glutathione (GSH)-responsive nanoplatform (TAF-HMON-CuP@PPDG) based on the hollow mesoporous organosilica NP (HMON). Generally, the overproduced endogenous GSH within tumor cells evokes the degradation of HMON, thus releasing tamoxifen (TAF) and copper peroxide (CuP). CuP can degrade into Cu^2+^ and exogenous H_2_O_2_ under acid stimulation, providing substrates for the consequent Fenton-like reaction catalyzed by Cu^2+^. Remarkably, the released TAF was able to enhance glycolysis and the subsequent lactate synthesis via suppression of mitochondrial complex I, thereby effectively promoting acidification in tumor cells, which could significantly facilitate the degradation of CuP to further augment H_2_O_2_ generation (Figure 2C) [36].

In addition to H_2_O_2_ and O_2_ generators, there are some other substances that can be delivered to tumor tissue as alternative substrates of ROS synthesis. For example, dihydroartemisinin, a derivative of artemisinin, contains an endoperoxide bridge, which can be cleaved by transition metals to produce highly cytotoxic •OH [86]. Accordingly, Chang’s group employed dihydroartemisinin in their study, where they designed a bovine serum albumin-tagged FeAl-LDH with a DHA cargo (BLD) as an effective cancer nanovaccine. Once the BLD is degraded to trigger a burst release of iron ions, aluminum ions and DHA, Fe^2+^ ions can catalyze both endogenous H_2_O_2_ and the released DHA, contributing to massive •OH synthesis in tumor cells. Compared to H_2_O_2_ alone, DHA involved dual sufficient substrates and provided better opportunities for the induction of ROS storm. Remarkably, the DHA can act as an immune regulator to suppress Treg cells by elevating IFN-γ levels, thereby enhancing immune responses in TME together with ICD caused by the ROS storm [87].

### Inhibit antioxidant systems

As we mentioned above, the antioxidant system is activated and develops a strong capability of ROS scavenging in tumors [88], which consists of enzymatic and non-enzymatic antioxidants. Non-enzymatic antioxidants mainly comprise GSH, CoQH_2_, bilirubin and some dietary compounds like vitamin C and vitamin E, while enzymatic antioxidants primarily include SOD, CAT, peroxiredoxin (PRX) and heme oxygenase-1 (HO-1), which play pivotal roles in antioxidant defense as well [89]. All of these collectively result in the formation of TME rich in antioxidants, making the tumor resistant to oxidative stress-mediated oncotherapy. Therefore, these antioxidants are considered promising targets to address this limitation.

Among the antioxidants mentioned above, GSH, an intracellular thiol-containing tripeptide, is the most plentiful non-enzymatic antioxidant molecule that is overproduced during oxidative stress and then extensively involved in ROS scavenging processes. Therefore, lowering GSH levels within tumor cells is a prospective strategy to inhibit ROS clearance [90]. Recently, multiple modalities based on this have been explored, which can be classified into two major subtypes: depleting GSH directly and interfering with the synthesis of GSH.

In terms of the GSH-consuming strategy, the most representative approach is to utilize exogenous oxidants to oxidize GSH via redox reactions, converting it to the disulfide form (GSSG). In this regard, substantial anti-cancer studies have employed disulfide-generating agents to elicit the disulfide–thiol exchange reaction, which have been confirmed to possess excellent capability of GSH depletion [91].

Beyond disulfide, a novel tetrasulfide has revealed to have a higher oxidation-reduction potential. In a recent example, Li *et al.* synthesized GSH-responsive virus-inspired hollow mesoporous tetrasulfide-organosilica (ssss-VHMS) containing NaCl nanocrystals within the interior (NaCl@ssss-VHMS). Once inside the tumor cells, the tetrasulfide linked to organosilica NPs could react with intracellular excess GSH via thiol-tetrasulfide exchange, which significantly eliminated GSH while initiating the degradation of ssss-VHMS, leading to the release of Na^+^/Cl^–^. The subsequent osmolarity surge would reinforce mitochondrial respiration, which facilitated the overproduction of endogenous ROS. Collectively, a strong ROS storm would be triggered. In addition, the osmolarity surge mediated by Na^+^/Cl^–^ would also cause direct cell swelling, which, in synergy with the ROS storm, leads to severe cell death including caspase-1-dependent pyroptosis, caspase-3-dependent apoptosis and GPX4-dependent ferroptosis [54].

**Figure 4:**
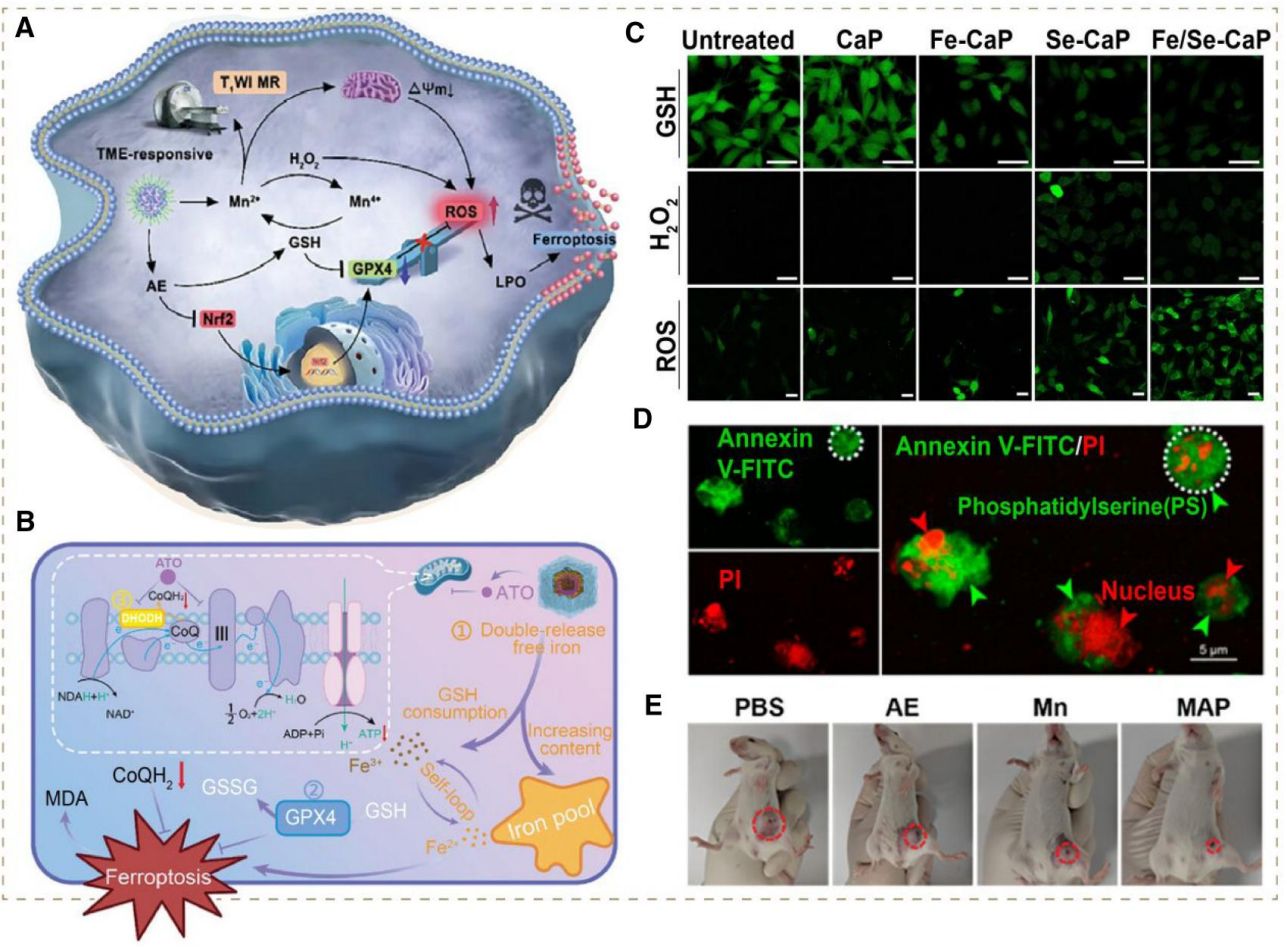
(**A**) Schematic illustration of the construction of the nanomedicine Mn-AE@PVP and its therapeutic mechanism. Reproduced with permission from Wiley-VCH GmbH, Copyright 2024 [52]. (**B**) Schematic of the strategy of AZFCP to induce ferroptosis of cascading storms with dual free iron release and breakdown of the ferroptosis-suppressing system. Reproduced with permission from Wiley-VCH GmbH, Copyright 2024 [59]. (**C**) GSH (green, probe: thiol track violet), H_2_O_2_ (green, probe: high-specific H_2_O_2_ probe) and ROS (green, probe: DCFH-DA) after various treatments. Reproduced with permission from Elsevier Ltd, Copyright 2021 [53]. (**D**) Apoptosis observation by CLSM in HepG2 cells treated with NaCl@ssss-VHMS using an annexin V-FITC and PI probe. Reproduced with permission from American Chemical Society, Copyright 2022 [54]. (**E**) Typical orthotopic 4T1 tumor-bearing mice showing the tumors in each treatment group after 14 days. Reproduced with permission from Wiley-VCH GmbH, Copyright 2024 [52].

Apart from polysulfides, high-valent metal ions exhibit potent GSH elimination capacity as well, some of which have already been employed in cancer therapies like Cu^2+^, Mn^4+^ and Fe^3+^ [92]. As a typical example, Yang’s group constructed polyvinyl pyrrolidone-modified self-assembling nanoscale coordination polymers based on manganese (II) acetate (Mn (CH_3_COOH)_2_) and 1,8-dihydroxy-3-hydroxymethyl-anthraquinone (aloe-emodin, AE) to synergistically induce dramatic ferroptosis. The released Mn^2+^ ions reacted with H_2_O_2_ generating •OH and Mn^4+^ via Fenton-like reaction. The generated Mn^4+^ subsequently directed a redox reaction with GSH, producing oxidized GSSG and Mn^2+^. Remarkably, compared with polysulfides, the transition metal ions could trigger a catalytic cycle in this process to achieve a synergistic effect of constant GSH depletion and CDT with a single agent. Eventually, the dramatic ferroptosis and further cancer eradication were induced significantly (Figure 4A and E) [52].

Like the high-valent metal ions with the capability of suppressing antioxidant systems and ROS synthesis simultaneously, most current studies focus on depleting excessive GSH to assist other ROS-based therapies instead of directly leveraging it to produce ROS. We hypothesize that the latter strategy can not only achieve valid GSH depletion but also alleviate the dependence on substrates for ROS synthesis. For instance, some compounds have been confirmed to possess the capability of catabolizing intracellular GSH directly to yield ROS through redox reactions. Among these, nano-Se has attracted increasing attention [93]. Peng’s group introduced a ferric ion and selenite-codoped calcium phosphate (Fe/Se-CaP) nanohybrid. The selenite component could produce highly toxic •$O_{2}^{-}$ and •OH by taking advantage of overproduced intracellular GSH as the substrate via cascade catalytic reactions, resulting in dual effects of ROS generation and antioxidant systems suppression (Figure 4C). In addition, the doped Fe could catalyze endogenous H_2_O_2_ into •OH, further adding to the ROS surge. Taken together, the ROS storm significantly promoted the activation of cleaved caspase-3 protein, downregulation of Bcl-2 protein and destruction of DNA in tumor cells, thereby inducing apoptosis-mediated production of TAAs and the subsequent stimulation of adaptive immune responses to inhibit tumor growth [53].

Moreover, GSH enzyme-like nanoenzyme is also widely employed, especially in the strategy of multi-nanoenzymes [94]. For instance, Zhao’s group constructed a copper-doping carbon nanozyme (CC) with multienzyme activity and integrated it with photosensitizer Ce6 and gelatin (CCC) to trigger ROS storms. In this process, upon the typical ROS production mediated by POD-like and OXD-like catalytic activity of multienzyme and photosensitizer Ce6, the nanozyme also exhibited the property of GSH OXD-like (GSHox-like) to effectively eliminate intracellular GSH, leading to abundant ROS accumulation [95].

Apart from the direct scavenging of GSH, disrupting GSH synthesis is also considered a potent strategy, which mainly comprises suppressing functional proteins in the GSH synthetic pathway and regulating gene expression.

**Figure 5:**
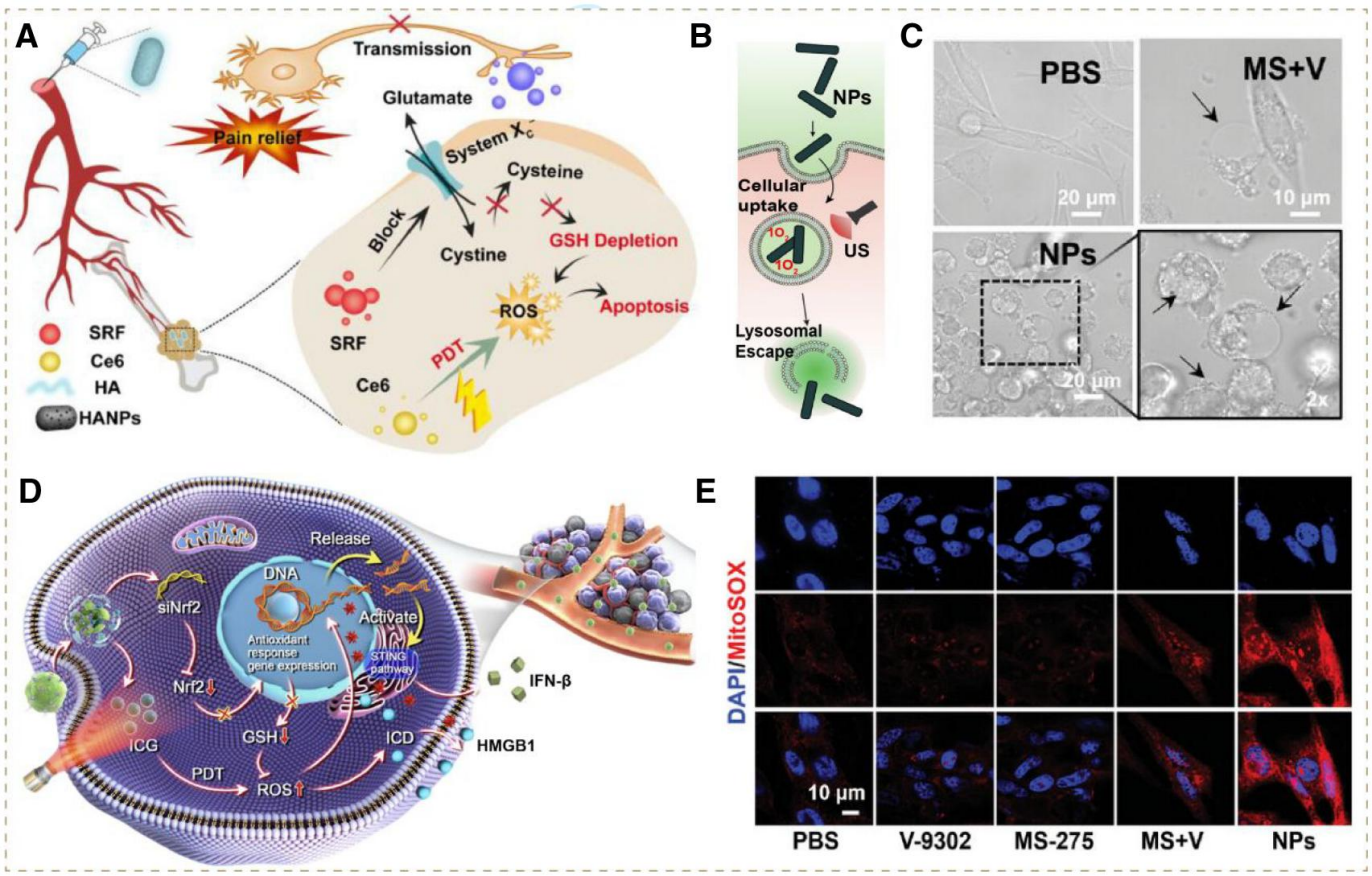
(**A**) Illustration of anti-tumor and analgesic effects of HA-SRF/Ce6@HANPs. Reproduced with permission from Elsevier, Copyright 2023 [56]. (**B**) Schematic illustration of ^1^O_2_-mediated lysosomal escape. Reproduced with permission from Elsevier, Copyright 2024 [57]. (**C**) Pyroptotic cells under microscope treated with PBS, MS+V and NPs for 24 h. Reproduced with permission from Wiley-VCH GmbH, Copyright 2024 [55]. (**D**) Schematic illustration of RI@Z-P-mediated amplification of oxidative stress and enhancement of immune response. Reproduced with permission from Elsevier, Copyright 2023 [58]. (**E**) CLSM images of mitoROS in cells. (blue, DAPI; red, MitoSOX). Reproduced with permission from Wiley-VCH GmbH, Copyright 2024 [55].

Glutamic acid, cysteine and glycine are indispensable substrates for GSH production, where their transporters, enzymes or other metabolic components can serve as potential disruption targets for decreasing GSH levels downstream. For example, Wang’s group prepared HYL001, a derivative of lonidamine, to downregulate glutaminase (GLS), limiting its function of catabolizing glutamine to glutamate, thus lowering intracellular glutamate concentration [96]. Additionally, Fu *et al.* utilized sorafenib to block cystine uptake via inhibition of the system Xc transporter (SLC7A11), which further led to cysteine reduction (Figure 5A) [56].

Under conditions of sufficient substrates, glutamate cysteine ligase (GCL) and GSH synthase (GSS) play critical roles in catalyzing the combination of the respective substrates to generate GSH. GCL can catalyze glutamic acid and cysteine into γ-glutamyl cysteine (γ-GC) with ATP consumption. Subsequently, GSS directs the construction of the linkage between γ-GC and glycine to generate GSH under ATP supply. During it, GCL acts as the key enzyme, making it a prospective target. For example, Li *et al.* designed a CaO_2_-based multifunctional nanocomposite with Cu^2+^ and L-buthionine sulfoximine (BSO) as the surface coating (named CaO_2_@Cu–BSO), reducing GSH production via GCL suppression by BSO. This synergistically enhanced the Fenton-like catalyst Cu^2+^-mediated CDT, leading to substantial accumulation of •OH within tumor cells [97].

Under oxidative stress, some regulatory pathways are activated in tumors to counteract ROS damage, like Nrf2-Keap1, HIF-1 and mTOR [89], which upregulate the transcription of genes encoding antioxidants and their biosynthetic enzymes. Generally, Nrf2-Keap1 plays the pivotal role, participating in the regulation of large-scale protein expression associated with antioxidants [98]. As outlined above, anti-oxidation pathways, especially the Nrf2-Keap1, stand as promising targets for weakening antioxidant systems of tumors at the genetic level.

Inspired by this, Sun *et al.* constructed a multifunctional nanoadjuvant (RI@Z-P) encapsulating Nrf2-specific small interfering RNA (siNrf2) and photosensitizer indocyanine green (ICG) with zeolitic imidazolate framework-8 (ZIF-8). Specifically, the expression of Nrf2 protein was downregulated via RNA interference by siNrf2, thus inhibiting the expression of genes relevant to antioxidants. Subsequently, the ROS generation mediated by the photosensitizer ICG under near-infrared (NIR) laser irradiation induced a robust ROS storm, which invoked ICD and led to severe DNA damage. DSB would result in the release of dsDNA into the cytoplasm where its accumulation could activate the transmembrane protein STING in ER, thus triggering a cascade reaction to promote IFN-β secretion. Taken together, ICD and the STING pathway synergistically intensified the immune response in TME by facilitating DCs maturation and stimulating T cells (Figure 5D) [58].

Compared with the Nrf2-Keap1 pathway, the mTOR pathway not only promotes glutamine uptake but is also extensively involved elsewhere in tumor progression, such as the regulation of nutrient metabolism and cell proliferation, thereby presenting a multifaceted therapeutic target. Notably, several mTOR inhibitors have already been employed in clinical use, which may offer a readily translatable opportunity to simultaneously suppress tumor growth and enhance oxidative stress. In a recent example, Ren *et al.* designed ROS-responsive NPs encapsulating HDACi MS-275 and the glutamine metabolism inhibitor V-9302. The HDACi MS-275 was employed to disrupt the mTOR pathway, while the V-9302 inhibitor could suppress the function of the glutamine transporter protein SLC1A5. Eventually, a robust ROS storm was triggered, inducing severe damage in the lysosome, mitochondrion (Figure 5E), and provoking dramatic pyroptosis (Figure 5C). The combination of transporter protein suppression and mTOR pathway interference collectively limited the glutamine uptake of tumor cells, thereby lowering intracellular glutamate concentration more effectively compared to the monotherapy of the mTOR inhibitor [55].

To date, most studies have focused on GSH scavenging, while other antioxidants also play important roles in tumor resistance to oxidative stress and may even increase in response to low GSH levels according to recent reports. Therefore, some studies have considered modalities to inhibit these antioxidants. For instance, dihydroorotate dehydrogenase (DHODH) mediates ROS detoxification by converting CoQ_10_ in the mitochondrial electron transport chain into reduced CoQH_2_, which possesses a prominent reducing ability to prevent lipid peroxidation. Inspired by this, Xu’s group utilized atovaquone (ATO) to inhibit DHODH in mitochondria, leading to diminished CoQH_2._ Additionally, the released transition metal ions induced reduction of GSH levels, further repressing GPX4 activity, leading to the enhancement of DHODH activity [59]. Furthermore, GPX4 could be directly suppressed by siGPX4, which was skillfully employed in the study conducted by Sun’s group, where they fabricated a TME-responsive MOF-based biomimetic nanosystem loading siGPX4 (mFeP@si) to inhibit GPX4 expression, resulting in the accumulation of highly toxic PL-OOH and the subsequent drug release from the lysosome (Figure 5B) and enhanced ferroptosis [57].

### Enhance catalytic efficiency

Presently, most nano-oncotherapies based on ROS storms are associated with chemical catalytic reactions [38]. Unfortunately, their anti-tumor effects can be hindered by insufficient catalytic efficiency to induce a lethal ROS storm. Therefore, apart from augmenting substrate supply and inhibiting antioxidant systems, intensifying ROS synthesis by increasing catalytic efficiency can be a potential strategy. Various factors impact catalytic reaction efficacy including the surrounding conditions, such as temperature and properties of the catalyst itself such as the size, specific surface area and active sites [99–101]. Accordingly, the current strategies can be primarily classified into two categories: optimizing the surrounding conditions during reactions and improving the catalytic properties of catalysts by adjusting the structure of nanodrugs.

In the former strategy, environmental conditions during catalytic reactions refer to conditions like temperature, pH and pressure. Therefore, it will be conducive to improving catalytic efficiency through these factors.

**Figure 6:**
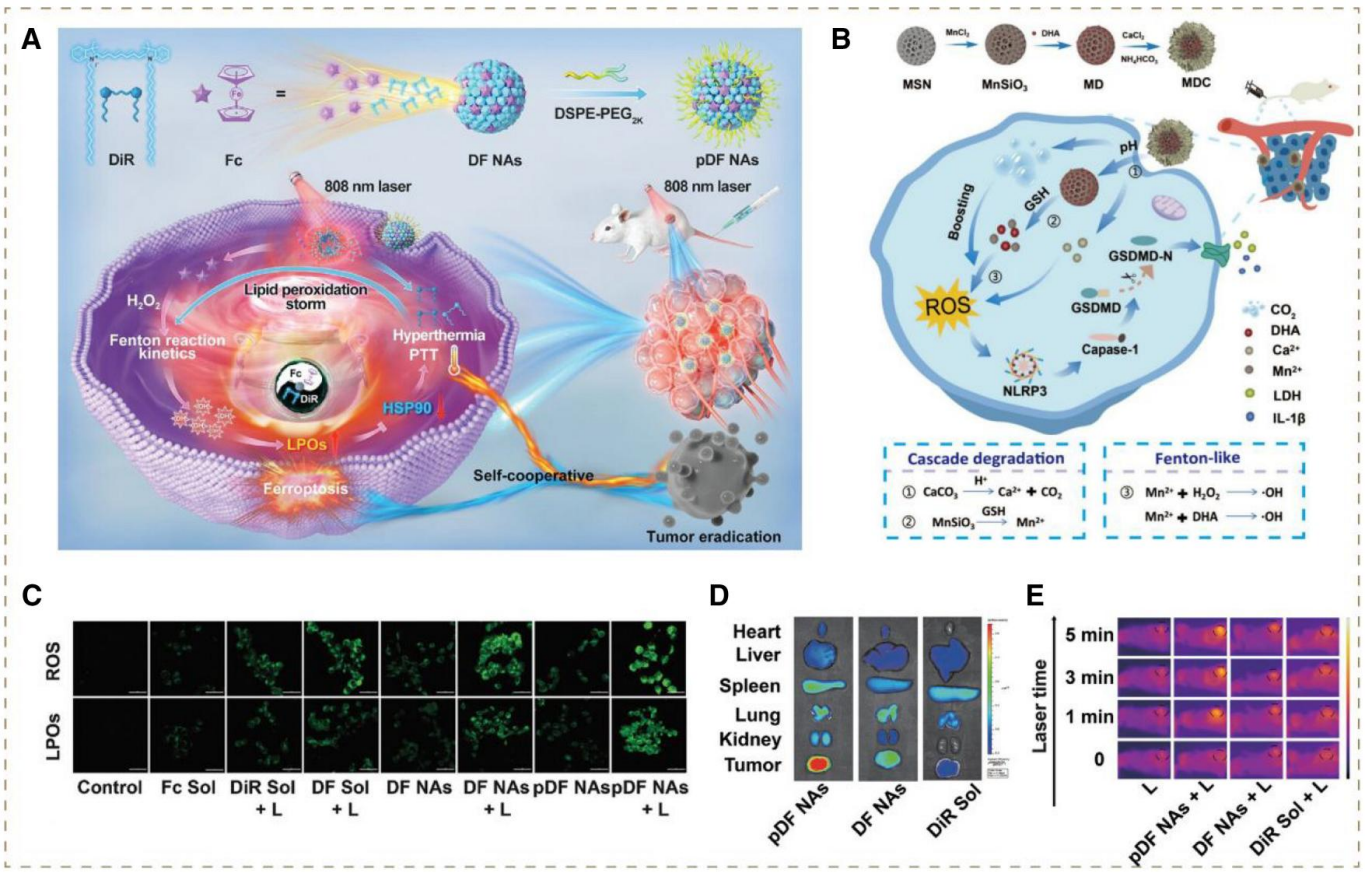
(**A**) Schematic presentation of a self-cooperative binary nanoassembly for imaging-guided photothermal/ferroptosis synergistic tumor eradication. Reproduced with permission from Wiley-VCH GmbH, Copyright 2024 [61]. (**B**) Schematic illustration of synthesis process of MnSiO_3_@DHA@CaCO_3_. Reproduced with permission from American Chemical Society, Copyright 2024 [62]. (**C**) Intracellular levels of ROS and LPOs in 4T1 cells after treatments, scale bar = 50 *μ*m. (**D**) *Ex vivo* images of major organs and tumors after 12 h injection of various formulations (*n* = 3). (**E**) *In vivo* photothermal images of 4T1 cells tumor-bearing BALB/c mice under 808 nm laser irradiation (2 W cm^−2^, 5 min) (*n* = 3). L: 808-nm laser. Reproduced with permission from Wiley-VCH GmbH, Copyright 2024 [61].

PTT is an anti-cancer treatment that increases the local temperature in tumor tissue to achieve cancer killing and exploit its vulnerability to chemotherapy and radiotherapy. Currently, the synergistic strategies of PTT and ROS storm have been widely explored to accelerate the catalytic reaction rate through high temperature [102]. Up to now, a number of novel nanomaterials as photothermal agents have been engineered to enable the above strategy like gold nanocages, magnetic Fe_5_C_2_ NPs, CuFeSe_2_ NPs and Nb_2_C Mxene nanosheets [69]. For instance, Wang and co-workers developed Pt nanocubes with Mn doping, which could significantly provoke photothermal ROS storm with synergistic strategies of PTT and CDT. The doped Pt possessed excellent photothermal conversion, elevating tissue temperature under 808-nm laser irradiation, thereby facilitating the efficiency of Mn^2+^ mediated Fenton-like reactions [60]. Yang and co-workers designed a self-cooperative nanomedicine (pDF NAs) based on molecular nanoassembly (NA) of DiR (a photothermal probe) and ferrocene (Fc, a reactant of the Fenton reaction) to provoke lipid peroxidation storm (Figure 6A). Firstly, the elevated high temperature facilitated the catalysis of the Fc-mediated Fenton reaction, producing considerable LPOs during ferroptosis (Figure 6C). Thereafter, the aldehyde degradation products of LPOs could degrade HSPs to weaken the heat resistance of tumors, thus enhancing the PTT therapeutic effect (Figure 6D and E). Overall, CDT induced lipid peroxidation storm and PTT enhanced each other’s efficacy for potent tumor-killing [61].

pH is also essential for catalytic efficacy, especially for Fenton and Fenton-like reactions. Each type of catalyst has a suitable pH range under which it exhibits maximum catalytic efficiency. For example, conventional iron-based Fenton catalysts work well at pH 2-4, while manganese-based Fenton-like agents are highly efficient at pH 5-6 [103]. Nevertheless, although TME is overall acidic compared with normal tissue, the intracellular pH (pHi) is higher than the extracellular pH (pHe) owing to the transporter-mediated efflux of H^+^ and is further complicated by the pHe heterogeneity from near-neutral to intensely acidic pH [104]. This TME can impair the catalytic activity of many ROS-generating agents, which underscores the significance of pH modulation.

Accordingly, Hu *et al.* designed an innovative MnSiO_3_@DHA@CaCO_3_ nanocatalyst (MDC). After entering tumor cells, the overproduced GSH could cleave manganese-oxygen bonds (-Mn-O-) in MnSiO_3_ NPs via oxidized/reduced reactions, evoking degradation of MDC, releasing Mn^2+^ and CaCO_3_. Under the acidic environment, CaCO_3_ would be degraded to Ca^2+^ and CO_2_ to elevate pH, increasing the efficacy of Mn^2+^ to yield •OH. Additionally, the dissolved CO_2_ could produce ${\text{HCO}}_{3}^{-}$, which coordinated with Mn^2+^ to form Mn-${\text{HCO}}_{3}^{-}$ complexes. These complexes stabilized the oxidation state of Mn^2+^ by preventing its precipitation while serving as electron transfer mediators, enabling a two-electron transfer pathway involving the Mn^2+^/MnIV=O redox couple and peroxymonocarbonate (${\text{HCO}}_{4}^{-}$) intermediates. Overall, CDT could be significantly enhanced, thereby initiating a robust ROS storm (Figure 6B) [62].

As discussed, suppressing the tumor’s antioxidant system prevents the clearance of therapeutic ROS, while supplying ample substrates provides the foundational reactants for their synthesis. To effectively convert these substrates into a potent ROS storm, a highly active catalyst is essential. This brings out the significance of intrinsic properties of catalysts, especially in nanomaterial-based therapies such as nanozyme strategies and photocatalysis, where nanotechnology allows for the practicality of adjusting the internal structure of catalysts [105, 106]. Among these, the specific surface area, micropores, morphology, chemical composition and size are all considered to be vital factors [101].

**Figure 7:**
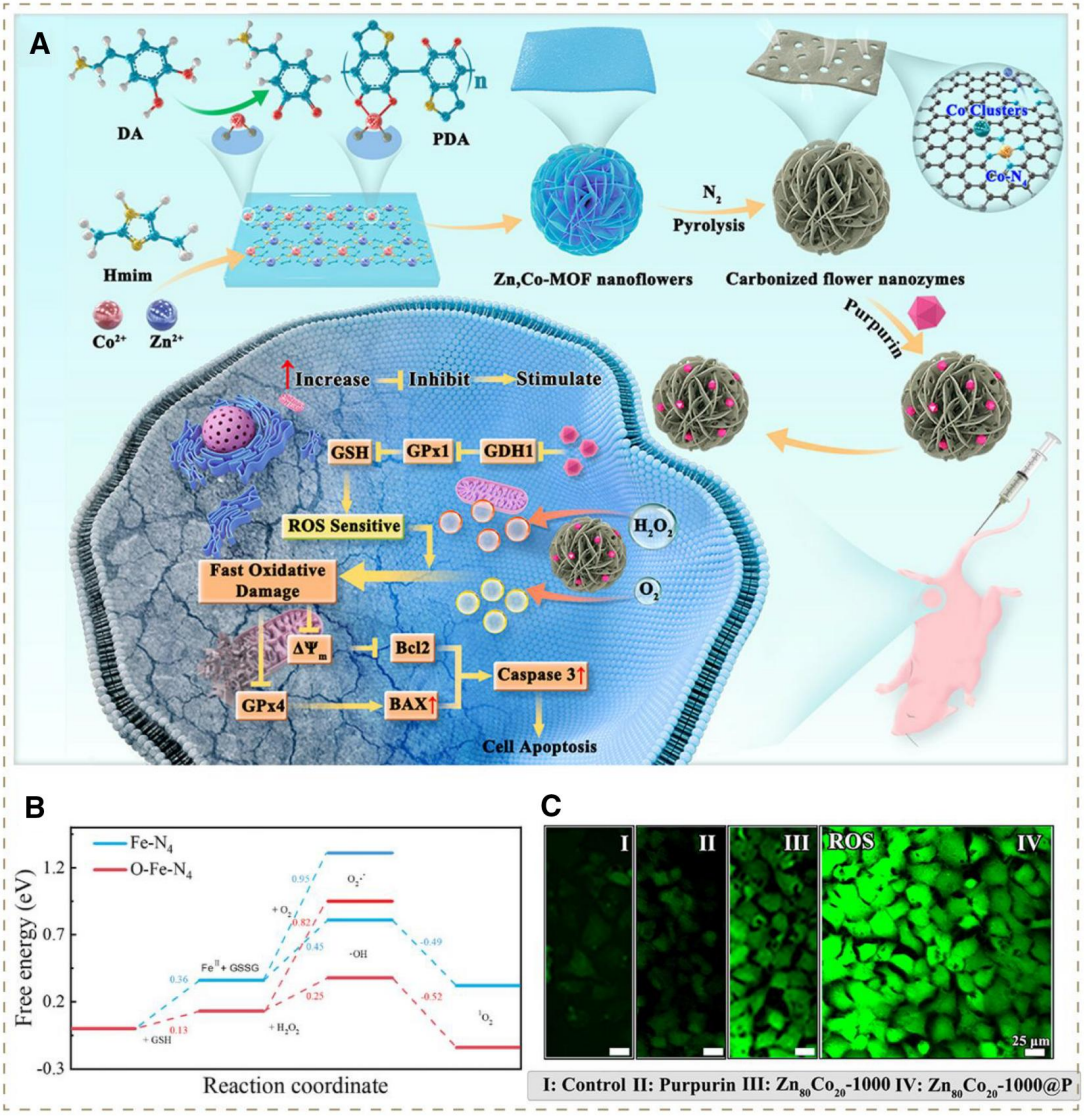
(**A**) Schematic illustrations of synthesis tactics of a ROS storm generator carbonized flower nanozymes. Reproduced with permission from American Chemical Society, Copyright 2024 [6]. (**B**) Corresponding free energy diagram of FeN_4_ moiety and O-FeN_4_ moiety. Reproduced with permission from Wiley-VCH GmbH, Copyright 2024 [63]. (**C**) CLSM images of ROS in cells after different treatments for 24 h. Reproduced with permission from American Chemical Society, Copyright 2024 [6].

The specific surface area is associated with the number of effective active sites of catalysts, while the micropore influences the diffusion efficiency of substrates. Various oncology treatment modalities concentrating on the optimization of them have been developed in recent years. For example, Xing and co-workers fabricated a flower-like nanozyme with a highly porous carbon matrix, which possessed high catalytic efficiency superior to conventional nano-/biozyme (Figure 7A). In detail, the ZIF was added with polydopamine (PDA), which underwent polymerization and large deposition on the porous leaflets of the nanoflower promoted by co-injected cobalt. The resultant concomitantly polymerized PDA could form a connected skeleton with excellent supporting capability, which could protect the porous leaflets from micropore shrinkage and skeleton collapse under the condition of pyrolysis during the preparation process. Eventually, it could increase the specific surface area and micropores of nanozymes, further accelerating their catalytic property for the rapid accumulation of ROS. In the meantime, the loaded purpurin drugs inhibited ROS elimination via down-regulating GSH expression (Figure 7C) [6]. Huang *et al.* constructed a versatile nanoplatform (designated as MCPQZ) based on high-density Cu_2_O-supported MoS_2_ nanoflowers. Specifically, Cu_2_O was densely loaded on photothermal MoS_2_ nanoflowers. Compared with previous studies, Huang’s group utilized copper glycinate for *in situ* growth of Cu_2_O on MoS_2_ nanoflowers, achieving higher Cu_2_O content and better monodispersity, thereby endowing MCPQZ with more catalytic sites for the subsequent Fenton-like reaction mediated by Cu ions [66]. Additionally, Liu’s group designed a novel efficient nanocatalyst carrier—platinum, where the ultrasmall Pt-based NPs were uniformly dispersed within a poly (amino acids) carrier (pGA). The ultrasmall size (mostly <5 nm) of Pt-based NPs significantly enlarged the specific surface area for more catalytic site exposure. Moreover, pGA acted as a structural matrix for Pt-based NPs, minimizing their aggregation through a defined porous framework. Taken together, the carrier-platinum exhibited superior activity of ROS production over iron and copper in tumors, which provided an innovative avenue for CDT catalysts in the future [67].

In the past decades, single atom nanoenzyme (SAE) has aroused widespread interest in the field of nanoenzyme-based oncotherapy owing to its maximum atomic utilization efficiency and excellent catalytic property [107]. However, the typical metal-N_4_ structure of traditional SAE failed to satisfy the requirements of ROS storm induction. Accordingly, emerging optimization strategies have endeavored to enhance the catalytic property of SAE by adjusting the original metal center. In a recent example, Liu and co-workers introduced the axial heteroatom O coordination in metal-N_4_ configuration to construct optimized carbon nitride quantum dots (O-Fe-N_4_), which had demonstrated superior catalytic activity (Figure 7B). Eventually, the innovative SAE could reinforce ^1^O_2_ surge via the Russell-type reaction. Meanwhile, the ROS loss mediated by GSH could be avoided with the GSH oxidase-mimicking activity of this nanodrug [63].

Semiconductor nanocatalysts have been extensively utilized in photocatalytic therapy due to their high stability. Their inherent bandgap structures enable the generation of electron-hole pairs (e-h^+^) upon photo-stimulation, then driving cascade redox reactions that magnify ROS generation. Typical semiconductor nanocatalysts primarily comprise TiO_2_ NPs, graphite nitride carbon nanosheets, black phosphorus quantum dots and others [108]. Unfortunately, the therapeutic effects of photocatalysts with traditional structures are unsatisfactory due to their insufficient catalytic efficacy. On these grounds, Liu’s group designed a reactor Fe/N_V_-CN. Based on graphite carbon nitride, they further modified it with nitrogen vacancies (N_V_) and ferric ions (Fe^3+^), which adjusted the band structure of CN via the quantum confinement effect and the altered coupled oscillations of atomic orbitals, thus endowing the catalyst with the capacity of oxidizing the accumulated by-product OH^−^ into highly toxic •OH. Moreover, the modification established dual carrier-transfer channels for electrons and holes to respective active sites through the introduction of stepped electrostatic potential and shortening three-electron bonds, leading to more carriers involved in •OH synthesis, thereby amplifying the efficiency in catalysis under illumination. Overall, on the basis of the intrinsic ability of CN to convert H_2_O_2_ into •OH, the structure adjustment augments •OH generation by utilizing accumulated by-product OH^−^ as the additional substrate, and optimizing the catalytic activity of the photosensitizer [64].

Heterojunction is another promising structure design to accelerate the reaction rate, which can augment the light utilization and retain the separation of photo-triggered electron-hole pairs [109]. Inspired by this, Gao and co-workers developed a nanocomposite (mUCC) based on the upconversion nanoparticle (UCNP) as the core and wrapped it with heterojunction CeO_2_/Cu_2_O. mUCC possessed dual catalytic means to generate ROS, comprising photocatalysis and Fenton-like reaction. Under the irradiation, the electrons and holes of the semiconductor were separated, transforming light energy into chemical energy, subsequently catalyzing oxygen, H_2_O or others to ^1^O_2_, •$O_{2}^{-}$ and •OH. Meanwhile, Ce (III)/Ce (IV) and Cu (II)/Cu (I) had the activity to initiate Fenton-like reaction. Based on the above-mentioned ROS-generating reactions, the heterostructure composed of two types of semiconductors improved the efficiency of catalytic reactions via enhancing the light response scope and facilitating the separation of electrons and holes, thereby preventing energy loss due to recombination through interface effects of heterostructures [65].

### Enrich the diversity of reactive species

To date, the majority of anti-cancer therapies based on oxidative stress have focused on the application of common ROS generation such as ^1^O_2_, •OH and •$O_{2}^{-}$ via biosynthesis with the employment of substances in cells [110]. However, their therapeutic efficiency can be restricted due to their reliance on TME. Moreover, the limitations of single ROS types can hamper the tumor-killing effects of oxidative stress. For instance, •OH is the most reactive species with strong cytotoxicity, which can oxidize most intracellular substances. However, the extremely short half-life (10^−10^ s) and the limited diffusion distance constrain its oxidative damage.

In addition to oxygen-centered ROS, there are other reactive free radicals with unpaired electrons localized on other atoms as well. They can be categorized into inorganic and organic radicals. Organic radicals refer to those carbon-centered radicals such as alkyl radicals, the generation of which is independent of substrate concentration, thus alleviating the restrictions of the TME. Inorganic radicals encompass nitrogen-centered reactive nitrogen species (RNS) like nitric oxide (NO) [111], sulfur-centered radicals such as sulfate radicals (•${\text{SO}}_{4}^{-}$) [112] and chlorine-centered radicals including hypochlorite radical (•ClO).

The radicals above differ in their sources, mechanisms of generation, diffusion distances and intracellular molecules with which they promptly react. Furthermore, some free radicals can react to generate new radicals [110, 113]. Accordingly, combining different types of radicals rationally stands as a potent modality to enrich accumulated reactive species in tumors to address the constraint of TME, avoiding limitations of single free radicals while leveraging upon each radical’s advantages. Ultimately, it can induce multifaceted damage on a large scale. Recent advances in nanotechnology have offered various means to achieve the strategy including multiple enzyme mimic nanoenzymes, nanoplatforms loaded with diverse radical generators, catalysts and others [114].

**Figure 8:**
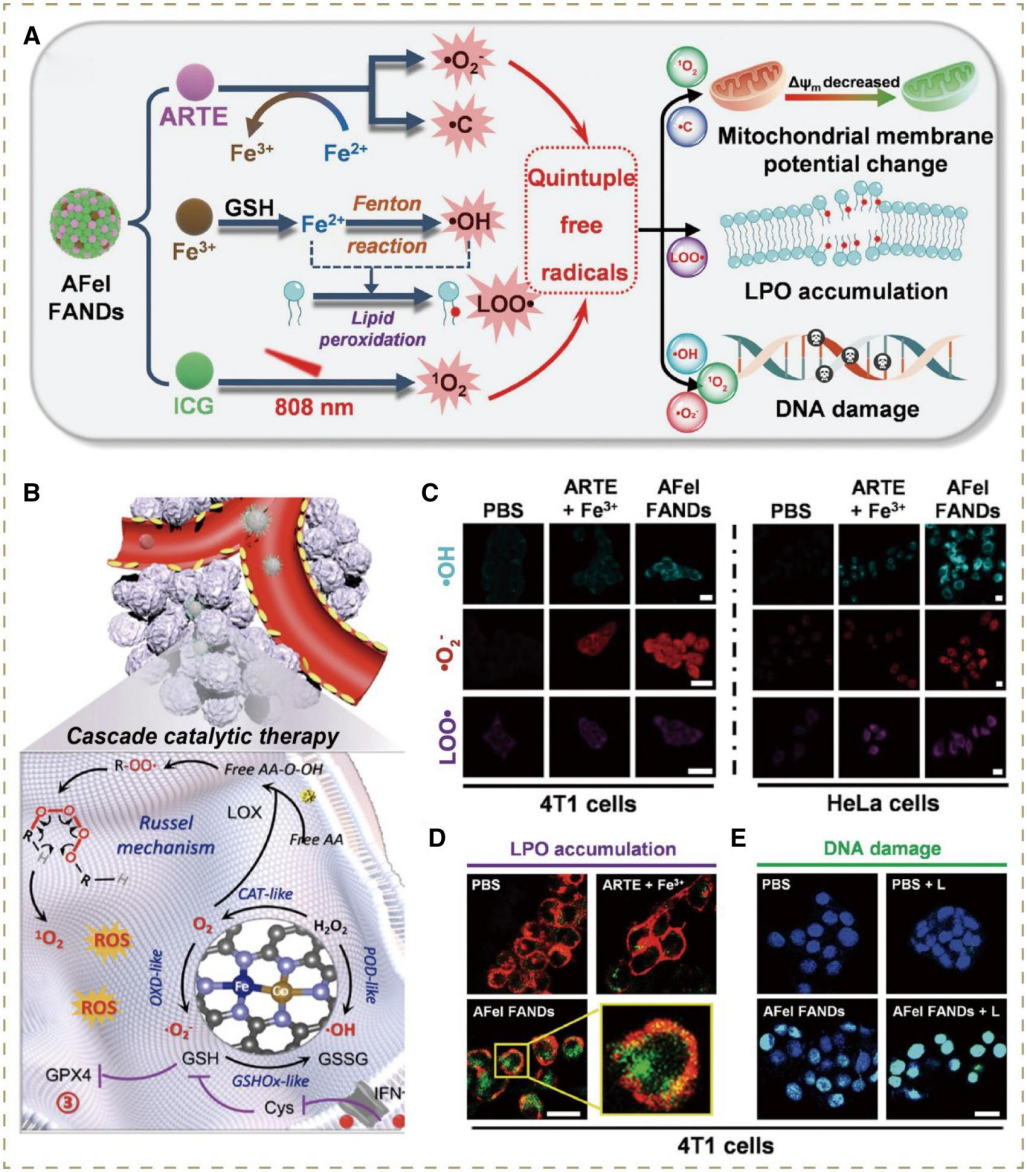
(**A**) Schematic illustration of five types of free-radical generation and multiple site-acting mechanisms of the AFeI FANDs. Reproduced with permission from Wiley-VCH GmbH, Copyright 2024 [70]. (**B**) Schematic illustration of the synthetic process and therapeutic mechanism of FeCo/FeCo DAzyme/PL. Reproduced with permission from American Chemical Society, Copyright 2023 [68]. (**C**) CLSM images of intracellular ROS generation (•OH, •$O_{2}^{-}$, and LOO•) after treatment with PBS, ARTE + Fe^3+^ and AFeI FANDs (•OH, LOO•, ex: 488 nm; •$O_{2}^{-}$, ex: 561 nm). scale bar = 20 *μ*m. (**D**) Cell membrane colocalization assessment of the LPO produced by the AFeI FANDs (green: LPO; red: cell membrane, ex: 640 nm). Scale bar = 20 *μ*m. (**E**) DNA damage in 4T1 cells after treatment with PBS, PBS + L, AFeI FANDs and AFeI FANDs + L (blue: nucleus, green: γ-H2AX, ex: 488 nm). Scale bar = 20 *μ*m. Reproduced with permission from Wiley-VCH GmbH, Copyright 2024 [70].

To date, a large number of multi-nanoenzymes with multiple enzymatic activities have been utilized, providing a promising strategy to produce diverse intracellular ROS via different catalytic means. As an example, Liu’s group designed multienzyme co-expressed dual-atom nanozymes (FeCo/FeCo DAzyme/PL), preparing FeCo/Fe-Co dual-metal atom nanozyme loaded with natural enzymes phospholipase A2 (PLA2) and lipoxygenase (LOX) to create a six-enzyme activity. On the one hand, PLA2 catalyzed phospholipids to release free arachidonic acid (AA), which was subsequently oxidized by LOX to form AA hydroperoxide (AA-OOH). Through the Russell mechanism, AA-OOH decomposed to generate ^1^O_2_. On the other hand, the dual-atom nanozyme possessed properties of four types of enzymes to produce various ROS. The activity of POD converted H_2_O_2_ into •OH, while the activity of OXD was able to transform O_2_ to •$O_{2}^{-}$. CAT mimetic activity could produce sufficient O_2_ by catalyzing H_2_O_2_, thus augmenting the substrate supply for catalysis mediated by PLA2, LOX and OXD. Taken together, multiple ROS storms composed of ^1^O_2_, •OH and •$O_{2}^{-}$ triggered irreversible immunogenic tumor ferroptosis via cooperation with ACSL4 activation mediated by IFN-γ (Figure 8B) [68]. Carrier-free nanodrug has become a promising candidate for disease theranostics owing to its distinct advantages of active pharmaceutical ingredient (API) loading, avoidable carrier-induced toxicity and convenient synthetic procedures [115]. Inspired by this, Zhang and co-workers designed a quintuple free-radical nanogenerator (AFeI FAND), realizing the assembly of multiple radical generators in virtue of the full-API nanodrug strategy [70]. Via the synergy of Fenton catalyzer Fe^2+^, •C generator ARTE and the photosensitizer ICG, AFeI FAND would raise a quintuple free-radical storm covering ^1^O_2_, •OH, •C, LOO•, •$O_{2}^{-}$ (Figure 8A and C), which could significantly elicit severe oxidative damage in mitochondria, DNA and cell membrane (Figure 8D and E).

**Figure 9:**
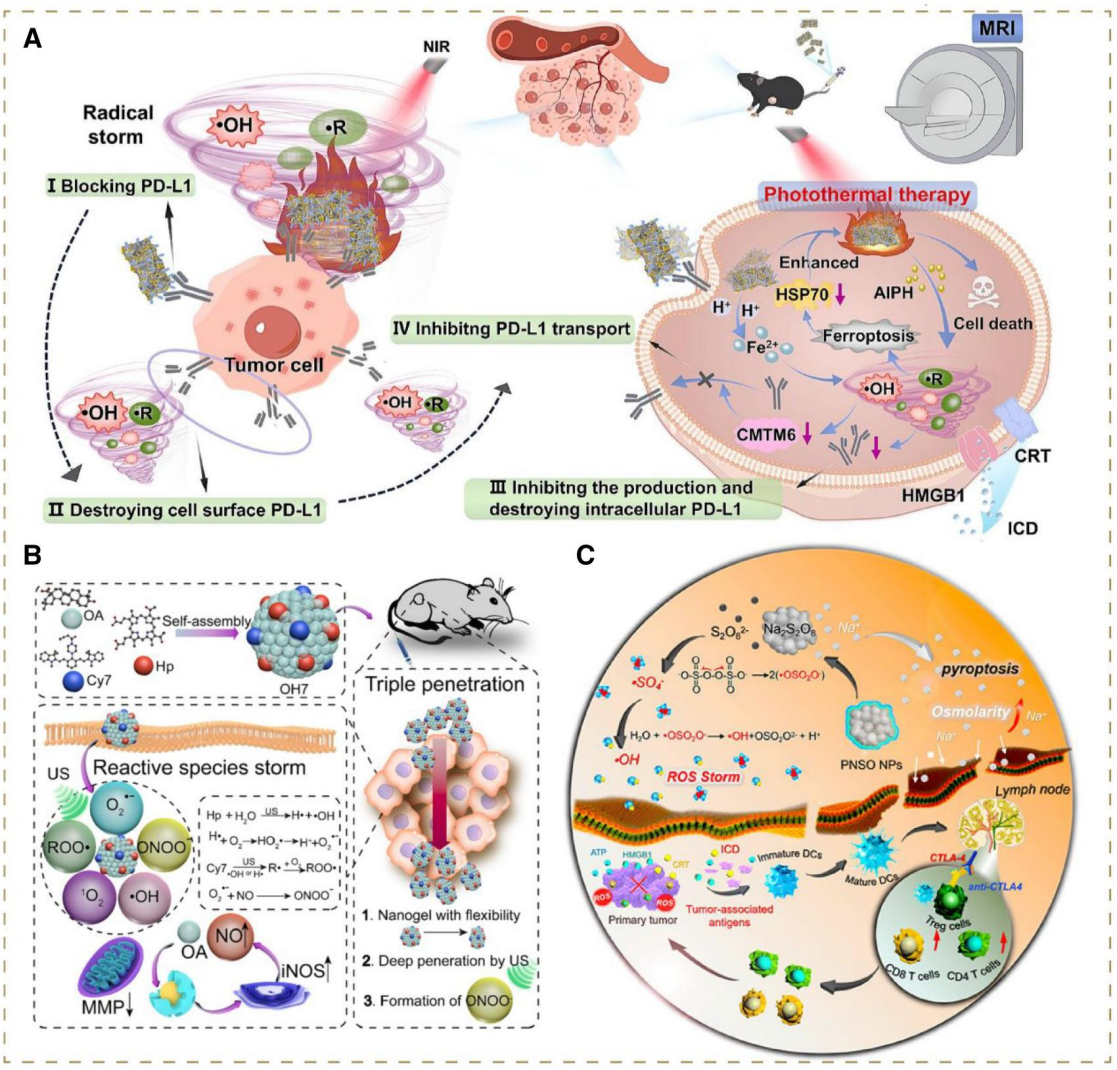
(**A**) Schematic illustration of the mechanism of multi-layered PD-L1 inhibition by ultrathin ferrous sulfide nanoplatforms with photothermal-triggered radical storm. Reproduced with permission from Elsevier B.V, Copyright 2025 [119]. (**B**) Self-assembled OH7 nanoreactor for ROO• and ONOO^−^-mediated sonocatalytic therapy and deep penetration for efficient cancer therapy. Reproduced with permission from Elsevier B.V, Copyright 2022 [71]. (**C**) Illustration of therapeutic mechanism of PNSO NPs. Reproduced with permission from American Chemical Society, Copyright 2020 [7].

Owing to the unique properties of other free radicals and the independence of TME in their generation process, numerous oncological treatment modalities combining ROS with other free radicals have emerged. As an example, alkyl radical generators have been widely utilized [116]. The most common generators primarily include 2,2’-Azobis[2-(2-imidazolin-2-yl)propane] dihydrochloride (AIPH), V057 and AIBA, which can be degraded to produce •C under heat or light stimulation. Moreover, some novel generators such as artesunate (ARTE) and anthracene diol possess the properties of photosensitizer and •C synthesis simultaneously [117, 118]. On these grounds, with an increasing progress made in nanoplatform construction, substantial researchers have started to co-load •C generators with other ROS generators or catalysts onto nanoplatforms, thus further enriching the diversity of oxidative species storm and enhancing the stability and biosafety of •C generators as well. To date, the synergistic modality of combining •C generators with PSs or photothermal agents has been one of the most common applications. The studies employing •C generators in collaboration with Fenton and Fenton-like catalysts have also emerged. In relation to this, our group constructed a novel ultrathin ferrous sulfide (FeS) nanoplatform loaded with AIPH and grafted anti-PD-L1 peptides (FAPP) for the first time to trigger a strong •OH/•R radical storm and achieve a tripartite PD-L1 blockade. During this process, upon NIR irradiation, FAPP served as a highly efficient photothermal agent to elevate local temperature, which magnified •OH generation via the acceleration of Fe^2+^-mediated Fenton reaction and initiated AIPH decomposition, releasing substantial •R. Taken together, the •OH and •R made up a robust radical storm, which effectively blocked PD-L1 in three dimensions: (i) directly destroying the PD-L1 on the surface of tumor cells; (ii) suppressing the generation of endogenous PD-L1; (iii) inhibiting the transportation of PD-L1 from the cytoplasm to the cell membrane via CMTM6 downregulation. A potent immunotherapy could thus be achieved in the synergy of PTT, •R/•OH storm and anti-PD-L1 peptides (Figure 9A) [119].

Alkoxyl (RO•) and peroxyl (ROO•) radicals are reactive organic radicals centered on the oxygen atom as well. ROO• possesses a long half-life and a large diffusion range, thus being able to achieve ultra-long tumor retention and multiple forms of damage. Moreover, lipoperoxide free radical (LOO•), one of the ROO•, is the key factor in tumor ferroptosis with its high toxicity [120]. Inspired by this, Li *et al.* developed an herbal medicine-based nanoreactor (OH7) self-assembled by oleanolic acid (OA) and two sonosensitizers (Figure 9B). The photo/sonosensitizer heptamethine cyanine (Cy7) and sonosensitizer hematoporphyrin (Hp) jointly induced consecutive reactions under sonication collaboratively to produce ROO• and •$O_{2}^{-}$. Moreover, OA activated endogenous NO synthase (iNOS) to generate NO, which reacted with •$O_{2}^{-}$ to produce long-lived free-radical peroxynitrite (ONOO^−^). Collectively, •$O_{2}^{-}$, ROO• and ONOO^−^ initiated highly toxic reactive species storms, inducing apoptosis of deep tumors [71].

Beyond organic radicals, RNS stand as potent inorganic radicals extensively employed in oncotherapy, which mainly include NO, nitrite (${\text{NO}}_{2}^{-}$), nitroso (HNO), nitrogen dioxide (NO_2_) and ONOO^−^. Similar to ROS, RNS play a dual role in tumors as well [113]. Notably, compared to most ROS, RNS exhibit superior oxidative properties and longer half-life. NO is the most typical RNS, which can induce nitrosative stress and regulate numerous physiological functions and pathological processes. Abundant NO generators have been fabricated ranging from exogenous generators classified into organic type (such as organic nitrates/nitrites, N-dicarboxylate, S-nitrosothiol and S-nitroso GSH, etc.) and inorganic type (such as metal nitroso), to endogenous NO synthases to inducing intracellular arginine production to NO. Various generators have been employed in direct tumor killing, TME recreation and inhibition of cell functions in collaboration with other therapeutic modalities. ONOO^−^ is also a potent RNS, distinguished from other ROS and RNS by its extremely strong cytotoxicity and its longer retention. Therefore, combining ONOO^−^ with ROS can prolong the duration of free-radical storms to enhance their cancer-killing effects. Inspired by this, Liu and co-workers constructed a nanoenzyme to induce reactive oxygen and nitrogen species (RONS) storms effectively. The released GOx could degrade intracellular glucose into gluconic acid and H_2_O_2_, which stimulated Fc by oxidation. The activated Fc could induce the Fenton reaction to produce •OH, while Arg was able to react with H_2_O_2_ via the catalyzer iNOS to generate NO. They both could be intensified by a sufficient H_2_O_2_ supplement via GOx. On this basis, numerous •OH could further be transformed into •$O_{2}^{-}$ via the Haber–Weiss cycle, which reacted with NO to generate ONOO^−^. Generally, the nanoenzyme could yield substantial •OH, •$O_{2}^{-}$, NO and ONOO^−^, which collectively formed a long-lived quadruple RONS storm for significant tumor elimination [72].

•${\text{SO}}_{4}^{-}$, a novel reported inorganic free radical, was originally utilized in the environmental field. This free radical is still rarely studied in oncotherapy instead [121]. It possesses an endogenous-substrate-independent synthesis process, superior diffusion distance, longer half-life (30-40 *μ*s) and more importantly, higher cytotoxicity compared with other radicals such as highly reactive •OH [122]. Therefore, employing •${\text{SO}}_{4}^{-}$ generators is an emerging way to strengthen free-radical storms. Generally, the commonly employed generators comprise peroxydisulfate (PDS: S_2_$O_{8}^{-}$) and peroxymonosulfate (PMS: ${\text{HSO}}_{5}^{-}$) with the stimulation of light, heat and metal ions such as iron, cobalt and copper in the pH range of 2-9. PMS is considered more easily activated than PDS due to its asymmetric structure, which has been utilized in a large number of studies [123]. For example, Liu’s group developed Na_2_S_2_O_8_ NPs (PDS NPs) coated with phospholipid (PNSO NPs) to prolong the stability in water. After entering the tumor cell, PNSO NPs would decompose into Na^+^ and S_2_$O_{8}^{-}$. The S_2_$O_{8}^{-}$ subsequently reacted with H_2_O to yield •${\text{SO}}_{4}^{-}$ and •OH, resulting in a robust radical storm, while the burst release of numerous Na^+^ could provoke cell osmolarity surge. Taken together, the synergistic strategies of the radical storm and the ion-interfering therapy caused dramatic mitochondrial damage, triggering apoptosis and pyroptosis. The resultant cell lysis promoted the release of TAAs, which reinforced the immune response by facilitating the maturation and activation of immune cells (Figure 9C) [7]. Moreover, some other synergistic modalities of co-loading •${\text{SO}}_{4}^{-}$ generators with Fenton and Fenton-like catalysts, sonosensitizers or PSs have gained ever-increasing attention as well.

### Intensify targeting accumulation of nanodrugs

Since the efficacy of ROS-based cancer therapy is confined to the short lifetime and the limited diffusion distance, localizing oxidative stress in subcellular organelles via organelle-targeted nanomaterials may circumvent these restrictions [75]. By far, the most commonly reported targets include mitochondria, ER, nucleus and ribosome.

The mitochondrion is critical in various physical functions such as energy metabolism [124], which makes it a central hub for ATP synthesis and the production of 90% endogenous ROS [125]. Therefore, it is also sensitive to oxidative stress. It has been reported that endogenous ROS generation and accumulation within mitochondria can even be amplified upon oxidative damage [126]. The double-layered membrane of the mitochondria displays a negative potential, which means nanodrugs with a positive charge can target it via the electrostatic interaction. On this basis, various cationic groups have been designed, among which triphenylphosphine (TPP) has been extensively employed [127], along with others such as targeting peptides and transition metal complexes. Up to now, researchers have combined mitochondria targeting groups with tumor treatment strategies such as PDT and PTT [128]. Moreover, the mitochondria targeting groups have also been applied in oncotherapy based on redox homeostasis disruption, which can magnify oxidative damage by enriching ROS generators in mitochondria, thus avoiding the disadvantages of ROS in short lifetime and limited diffusion. Recently, great progress has been made in this strategy, which involved leveraging upon mitochondrial characteristics to provoke strong reactive species storms in mitochondria compared with previous studies.

**Figure 10:**
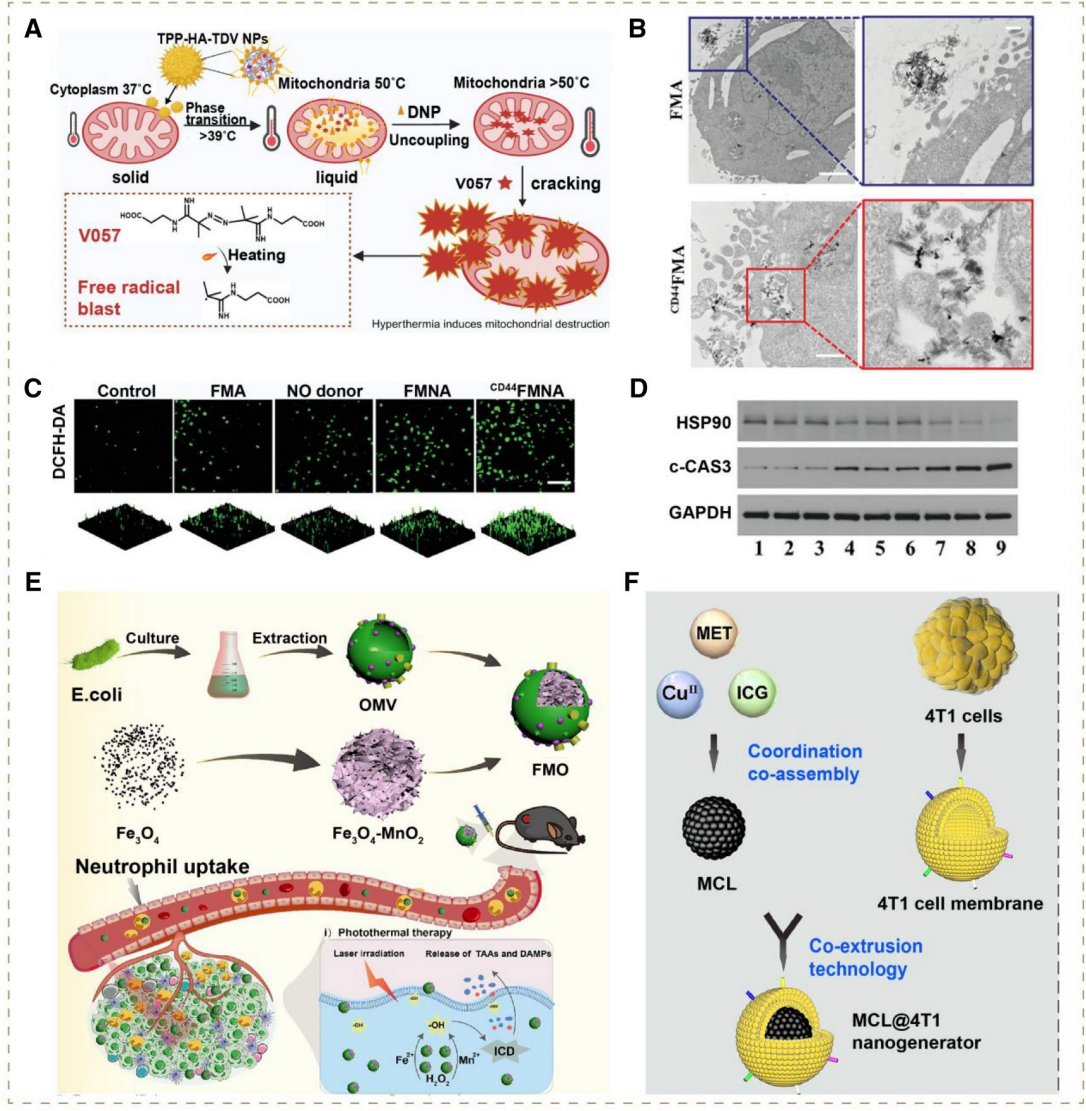
(**A**) Schematic illustrations of TPP-HA-TDV NPs induced immunogenic death and enhanced anti-tumor ability. Reproduced with permission from Elsevier, Copyright 2024 [75]. (**B**) Bio-TEM images of MDA-MB-231 cells co-cultured with FMA and ^CD44^FMA for 4 h, respectively. (**C**) Fluorescence images of the treated MDA-MB-231 cells with various formulations stained using DCFH-DA. Scale bar: 200 *μ*m. Reproduced with permission from Wiley-VCH GmbH, Copyright 2024 [73]. (**D**) Western blot analysis of HSP90/GAPDA and c-CAS3/GADPH protein in mice of different treatment groups. Reproduced with permission from Wiley-VCH GmbH, **C**opyright 2023 [32]. (**E**) Schematic illustration of the synthesis and therapeutic mechanism of FMO NPs. Reproduced with permission from American Chemical Society, Copyright 2023 [134]. (**F**) Schematic illustration of the fabrication process of MCI@4T1 via coordination co-assembly and cell membrane co-extrusion. Reproduced with permission from Elsevier, Copyright 2023 [135].

Most •R generators depend on heat stimulation to decompose and then release the radicals. Previous studies have reported that mitochondria reach a higher temperature (near 50°C) than the cytoplasm. Inspired by this, Peng *et al.* synthesized mitochondrial thermosensitive TPP-hyaluronic acid (HA)-TDV NP. Specifically, after the drug was targeted into the mitochondria, which could be observed via loaded methylene blue as a fluorescence probe, the phase-change material [tetradecyl alcohol (TCA)] caused the drug to transform from solid phase to liquid phase under the self-heat of the mitochondria, thus releasing the decoupler 2,4-dinitrophenol (DNP), which further amplified the heat inside mitochondria. The resultant high temperature within mitochondria served as a strong stimulus to evoke thermal decomposition of 2,2′-azobis[N-(2-carboxyethyl)-2-methylpropylamine] hydrate (V057, TDV), yielding numerous •C (Figure 10A) [75]. Consistent with this mechanism, the CCK-8 assay and flow cytometry (FCM) demonstrated a superior tumor-killing effect of TPP-HATDV than TPP-HA-TV, with the apoptosis ratio determined to be ∼34.31%, which indicated that with DNP raising the mitochondrial intrinsic temperature to trigger V057 initiation, more free radicals were released to cause cell damage. Overall, with the help of targeting groups, the magnified thermal effect via mitochondrial self-heat and decoupler greatly promoted •C synthesis by exploiting the mitochondrial property.

The ER is one of the most critical organelles, which is responsible for the biosynthesis, folding, modification and transfer of secreted and transmembrane proteins [129]. ER stress has been reported to be involved in the induction of cancer death as a vital signaling pathway, which is associated with the immune response stimulation as well. Therefore, enrichment of ROS generators in ER to evoke severe ER stress might be a potent avenue to enhance cancer eradication of ROS storms, especially in synergy with immunotherapy [130].

However, the efficiency of ER-targeted strategies can be compromised by the organelle’s adaptive and defensive responses. To overcome this, simultaneous targeting of multiple subcellular organelles has emerged as a promising approach. In this context, polymers with inherent organelle-disrupting capabilities have attracted increasing interest. Unlike traditional nanocarriers that require drug loading, these ‘drug-free’ macromolecules can induce organelle dysfunction and structural damage without incorporating conventional pharmaceutical agents, thereby avoiding limitations such as encapsulation efficiency and premature release.

Inspired by this, Wang *et al*. formulated an ER and mitochondria dual-targeting NPs (EMT-NPs) from P(ERMA-co-DMA)-b-PCSMA. The precise dual-targeting enhanced polymer binding to both organelles, leading to synergistic dysfunction of the ER and mitochondria. Typically, it triggered Ca^2+^ leakage from ER into cytoplasm and mitochondria, which subsequently amplified mitochondrial damage and boosted the release of endogenous ROS. Consistently, the total intracellular ROS level and the superoxide level in mitochondria were monitored via DCFH-DA and MitoSOX Red, respectively, which demonstrated an improvement in ROS generation with the incorporation of the ER targeting unit. The tumor-killing effectiveness evaluation against A549 cells also showed that the apoptotic ratio of EMT-NPs was determined to be ∼50.36%, and that of MT-NPs was ∼28.47%, indicating that ER and mitochondria targeting potently intensified cell damage [15].

While all the ROS storm strategies above are promising to provoke intense oxidative damage, whether the ROS generators can exactly accumulate in tumor sites is one critical premise that cannot be ignored. The limited target specificity of ROS generators may result in off-target biodistribution and non-selective cellular internalization, which may weaken the therapeutic effects without sufficient drug accumulation in tumor sites to trigger ROS storms and elicit biosafety issues. To overcome this challenge, researchers have sought to address this issue by designing multiple nanoplatforms with tumor-targeting capability to achieve precise delivery to the tumors. As a whole, enriching the specific accumulation of nanomedicine in the tumor tissue is identified as a potential strategy. So far, EPR passive targeting, biomolecule markers targeting and homologous targeting are the most extensively employed modalities in ROS-based anti-cancer therapy.

CD44 protein is a transmembrane glycoprotein on the cell surface, which is one of the most common biomarkers associated with breast cancer. Numerous studies have revealed that the expression level of CD44 protein in breast cancer cells is significantly higher than that in normal mammary cells, making it a promising target [131]. According to this, Wang’s group constructed a trimetallic nanoplatform with a conjugated targeting ligand anti-CD44 mAb (Fe_3_O_4_@MnO_2_@NO@Au NPs, abbreviated as ^CD44^FMNA). Consistently, the results of the experiments encompassing an inverted fluorescent microscope (IX73, Olympus) and Bio-TEM demonstrated that the loading of targeting ligand anti-CD44 mAb significantly intensified intracellular uptake of nanodrugs, with more ROS detected within cancer cells (Figure 10B and C) [73].

Targeting strategy based on peptides also possesses its unique advantages such as excellent tissue penetration capacity, decreased immunogenicity and a high affinity to interact with targets [132]. Integrin receptors are one of the representative peptides that are closely related to cancer progression. Remarkably, integrins can be highly upregulated in multiple tumors while remaining at a low or undetectable level in most normal epithelia, displaying their potential as therapeutic targets [133]. Inspired by this, Ding *et al.* constructed the nanosystem based on Cu^2+^ and ICG loaded PDA nanosphere and then modified it with integrin-targeted cyclic peptide (cRGD) (PDA/Cu/ICG/R) to achieve potent tumor elimination via synergistic therapy of mild-PTT, PDT and CDT. The surface modification of integrin targeting cyclic peptide could effectively magnify drug accumulation in breast cancer, which could be observed via the evaluation of CLSM and FCM. Thereby, compared with the PDA/Cu/ICG without cRGD, the PDA/Cu/ICG/R group modified with cRGD was detected to significantly elevate the level of ROS generated by the photosensitizer ICG and the Fenton-like catalyst Cu^2+^ due to the enhanced cellular internalization. Ultimately, the triggered ROS storm could amplify intracellular oxidative stress and downregulate HSP expression via constraining ATP synthesis through mitochondrial destruction, thus enhancing the anti-cancer effect of mild-PTT (Figure 10D) [32].

Nonetheless, those targeting strategies based on the modification of specific ligands can be imprecise and suboptimal in some cases. Recently, cell membrane coating technology has emerged as a promising method for homologous targeting. In contrast to ligand-based approaches, it exhibits superior tumor-targeting capability and inherits unique biological functions from the source cells, such as the capacity for immune evasion. This has provided an innovative approach for precise drug delivery. Cell membranes of red blood cells, bacteria, cancer cells, macrophages and immune cells have been widely exploited in oncotherapy. Among these, the cancer cell membrane coating is especially promising due to its capacity to prolong the blood circulation of drugs, reduce macrophage phagocytosis and selectively target homologous tumors (Figure 10F). On these grounds, Lei and co-workers fabricated a US-responsive MOF nanosystem (MFePCN@1-MT). In short, the tumor cell membrane camouflaged nanosystem could target and accumulate in osteosarcoma, which was evaluated by CLSM. Subsequently, FePCN yielded ROS via PDT and CDT. Notably, in K2M7 cells incubated with tumor cell membrane-modified nanosystems, a significant elevation in ROS production was observed compared with unmodified nanosystems, validating the ability of homologous targeting in augmenting ROS storm [74]. Other than cell membrane coating technology, the cell-hitchhiking strategy, mostly relying on either cellular internalization or covalent surface conjugation, is also a promising modality for the precise delivery of ROS generators. On this ground, Liu’s group constructed a drug-free bacteria-derived outer membrane vesicle (OMV)-functionalized Fe_3_O_4_-MnO_2_ (FMO) nanoplatform, where the drug accumulation in tumors was intensified via neutrophil-hitchhiking delivery after the modification of OMVs extracted from *Escherichia coli* (Figure 10E). It was observed via the examination of cellular uptake efficiency by CLSM. It is worth mentioning that with neutrophil equipment, the drugs could even be further enriched once the inflammatory response at the tumor site was triggered by PTT [134].

Collectively, the strategies discussed above have their advantages and shortcomings, respectively, while demonstrating close interrelationships. First, ample substrate supply is the fundamental premise for ROS synthesis. This strategy is particularly effective for tumors with a hypoxic TME or limited intracellular H_2_O_2_. However, the precise delivery of nanodrugs for this strategy remains a major challenge. Furthermore, even with sufficient substrates, efficient catalysts are still required to meet the high demand for ROS generation.

On this ground, potent catalysts are pivotal for amplifying ROS production. By accelerating the reaction kinetics, catalytic optimization can increase the concentration of ROS in a short time and prolong their duration via cascade reactions, thus compensating for the short half-life of ROS. Nevertheless, challenges such as the complex synthesis of nanocatalysts and their long-term biosafety can hamper the clinical use of this strategy. Moreover, the limited tissue penetration depth of external stimuli that facilitate the reactions is another barrier that needs to be conquered.

Suppressing antioxidant systems inhibits tumor cell-mediated ROS clearance, serving as a powerful adjuvant to other strategies mentioned above or to traditional chemotherapy. However, the application of antioxidant inhibitors can cause severe damage to normal tissues, which brings out the need for highly accurate tumor targeting.

The combination of multiple types of ROS and other reactive species broadens the range of oxidative damage to multiple positions in tumor cells, from DNA and mitochondria to the ER and the cell membrane. Meanwhile, it can also overcome the inherent limitations of individual species, such as their short diffusion distances. Unfortunately, the complexity of these multi-reaction systems poses significant challenges for the precise control of species generation and the fabrication of the corresponding nanoplatforms.

Additionally, subcellular organelle targeting overcomes the diffusion limitations of ROS by concentrating them at specific sites within tumor cells, thereby efficiently intensifying localized damage to subcellular organelles. While tumor cell targeting can intensify the accumulation of nanodrugs at the tumor tissue, confining the ROS storm to malignant tissue. This spatial control maximizes anti-cancer efficacy while minimizing off-target damage to healthy tissue.

In conclusion, achieving a robust ROS storm is difficult with any single modality due to the distinct features and limitations of each strategy. Therefore, a synergistic approach may stand as a promising path forward. Accordingly, continuous progress in nanotechnology has accelerated the development of multifunctional nanomaterials possessing both high loading capacity and multiple enzyme-like activities. Combining photothermal, sonodynamic and enzyme-mimetic catalytic therapies with novel ROS-generating agents is expected to yield a ‘1 + 1 > 2’ effect. For instance, ultrasound in SDT can enhance the catalytic rate of Fenton-like reactions. Likewise, piezoelectric sonosensitizers can reduce high-valence metal ions to their active low-valence states via electron transfer, thereby promoting greater ion participation in catalytic cycles.

## Conclusions, discussions and outlook

Redox homeostasis, regulated by the intracellular ROS generation system and antioxidant system, is of pivotal significance in ensuring the functionality and survival of cells. The imbalance of redox status is closely related to various diseases. In tumors, the redox status is aberrantly maintained, with both ROS and ROS scavengers overproduced, which is relevant to tumorigenesis, progression and metastasis. Therefore, one notable ROS-based oncotherapy strategy is aimed at disrupting this homeostasis by increasing ROS in tumor cells. Traditional ROS-based therapy is primarily centered on enhancing ROS generation, as seen in the optimization of dynamic therapy. However, the therapeutic efficacy is not solely a function of initial ROS levels, but is often constrained by several factors that have long been neglected. First, the characteristics of various ROS such as the lifespan, diffusion distance and the distribution within cells largely determine the extent of oxidative damage once they are generated intracellularly. Second, ROS play sophisticated and dual roles in the biological activity, directly and indirectly influencing tumor metabolism, signaling pathway and TME formation, thus affecting the tumor response to ROS-mediated therapy. Fortunately, recent advances in nanotechnology have offered strategies to address these challenges, thereby comprehensively amplifying oxidative damage, which we termed the ‘ROS storm’. This term is intended to encapsulate a synergistic multi-mechanism approach that moves beyond mere concentration increase. In this review, we systematically categorized and analyzed how these innovative nanomaterials are uniquely applied to break these bottlenecks.

Despite abundant studies with encouraging progress emerging in this field, there are still several issues that need to be addressed for the future development of the ROS storm modality based on nanotechnology. First, the biosafety profile of nanomaterials, including biocompatibility and biodegradability, is critical for the translational success of the aforementioned strategies. The unintended accumulation of nanodrugs in healthy tissues including the spleen and liver may lead to unexpected organ-specific toxicity and result in more sophisticated long-term toxicity. So far, clinically approved nanomaterials predominantly consist of organic materials such as liposomes that are biodegradable and nontoxic. Therefore, it is crucial to incorporate more natural materials into nanomedicines such as hybrid nanostructures, the surface modification of polymers, copolymers and biological agents [136, 137]. Second, the therapeutic mechanism itself introduces complex risks. ROS have different effects on tumor cells and normal cells at different levels. A low to moderate level of ROS can accelerate tumor growth and survival by acting as signaling molecules. It should also be noted that limited ROS can cause DNA damage in tumor cells. If this damage is insufficient to eliminate tumors, it may instead give rise to mutations that induce drug resistance [89]. In contrast, excessive ROS are more likely to cause impairment to normal tissue and lead to functional suppression or even severe damage to immune cells. Evidence indicates that T-cell function can be restricted by NO production and ROS release from tumor cells [138]. Apart from these, the impact of varying dosages of ROS on tumors differs across diverse tumor types and during different stages of tumorigenesis, from neoplastic transformation to invasive carcinoma. This complex regulatory mechanism makes the dosage control of ROS storm therapies and the precise targeting strategy crucial for their clinical translation. Third, current nanomaterials mostly utilize their EPR effect to achieve drug transport to tumors. However, whether the EPR effect is sufficient to enrich most of the drugs to tumor cells is less discussed during therapeutic strategies and designs. The EPR effect is prominently based on the elevated permeability of tumor vasculature and prolonged blood circulation of drugs with a nano size. Nevertheless, this tumor’s abnormal vasculature is closely associated with the leaky endothelial cell barrier and other abnormalities, which show heterogeneity within individual tumors and between various tumor types [40]. Additionally, the extracellular matrix, perivascular tumor cells and other features of TME may give rise to a barrier against nano medicine penetration [139]. Moreover, although the EPR effect has been validated by substantial research in mouse models, the volume of tumors in humans is higher than that of mice, which means the targeting efficacy of the EPR effect may still need to be potentiated for clinical translation. In fact, there have been studies making quantitative comparisons between this passive targeting and active targeting such as homologous targeting that demonstrated the tumor penetration of the EPR effect was relatively insufficient [140].

Based on the above problems, we offer a series of potential solutions aimed at minimizing side effects and expanding efficacy. First, the development of controlled ROS storm generation strategies may have the ability to address the above issues. For example, the timing of ROS generation can be controlled by using drug-release strategies that respond to the specific microenvironment of the tumor, or by using the ‘on and off’ strategy by employing the external stimuli as a switch to activate ROS generation. Catalytic reactions that yield ROS or substrates can be triggered by light irradiation and accelerated by photothermal energy. The utilization of external stimuli provides a way of identifying the optimal wavelength and intensity required to generate ROS at an optimal dosage [141]. Additionally, real-time monitoring of drugs using a range of imaging tools, such as photoacoustic imaging, allows for a better determination of the dose and position of drugs. This strategy can be implemented by nanoplatforms designed with sites for loading fluorescence probes, such as fluoresceinamine (FA) and cyanine5.5 (Cy5.5), to detect free radicals [142]. This strategy will enable real-time, *in vivo* quantification of ROS flux, variety and location, moving the field from a phenomenological to a mechanistic understanding of oxidative stress and its consequences. However, implementing this strategy places high demands on the optimal nanomaterials and nanotechnology during fabrication. The precision of fluorescence probes presents another challenge, considering multiple types of ROS and their short lifespans, especially *in vivo*. Hence, more exploration in the suitable nanomaterials, convenient production and accurate probes is a critical task for future research. Second, the synergy of ROS storms and immunotherapy is an important direction of the current research. First, ROS storms are potent inducers of ICD such as pyroptosis. This process promotes the release of TAAs, DAMPs and IFN-β in tumor tissues, which collectively facilitate DC maturation and antigen presentation, thereby initiating a robust adaptive immune response. Beyond ICD, ROS storms can remodel the immunosuppressive TME to intensify T lymphocyte infiltration and function. For instance, some ROS-generating strategies can disrupt tumor metabolism, thus depleting ATP and down-regulating immunosuppressive metabolites like lactic acid, which otherwise may impair immune cell activity [143]. Most notably, ROS storms offer a unique strategy to overcome the limitations of ICIs. They can not only cause the direct destruction of existing ICs on the tumor cell surface but also suppress the intracellular synthesis and transport of these proteins to the membrane. These mechanisms create a highly favorable condition for ICIs to achieve a complete and durable therapeutic blockade. It is also worth mentioning that various other types of reactive species are promising for tumor eradication as well. However, the current research on them is still limited. Therefore, we anticipate a shift beyond ROS to include other reactive species such as RNS, carbonyl species and •${\text{SO}}_{4}^{-}$, exploring their unique cytotoxicity and synergistic potential with ROS to create a more comprehensive ‘reactive species storm’.

## Data Availability

Data sharing is not applicable to this article as no datasets were generated or analyzed during the current study.
